# Noise Minimization in Cell-Free Gene Expression

**DOI:** 10.1021/acssynbio.3c00174

**Published:** 2023-07-21

**Authors:** Mart W. Bartelds, Óscar García-Blay, Pieter G. A. Verhagen, Elise J. Wubbolts, Bob van Sluijs, Hans A. Heus, Tom F. A. de Greef, Wilhelm T. S. Huck, Maike M. K. Hansen

**Affiliations:** † Institute for Molecules and Materials, Radboud University, Heyendaalseweg 135, 6525 AJ Nijmegen, The Netherlands; ‡ Laboratory of Chemical Biology, Department of Biomedical Engineering, Eindhoven University of Technology, P.O. Box 513, 5600 MB Eindhoven, The Netherlands; § Institute for Complex Molecular Systems, Eindhoven University of Technology, P.O. Box 513, 5600 MB Eindhoven, The Netherlands; ∥ Computational Biology Group, Department of Biomedical Engineering, Eindhoven University of Technology, P.O. Box 513, 5600 MB Eindhoven, The Netherlands; ⊥ Center for Living Technologies, Eindhoven-Wageningen-Utrecht Alliance, 5600 MB Eindhoven, The Netherlands

**Keywords:** cell-free gene expression, transcription and translation, microfluidics, gene expression noise, MazF, mRNA degradation

## Abstract

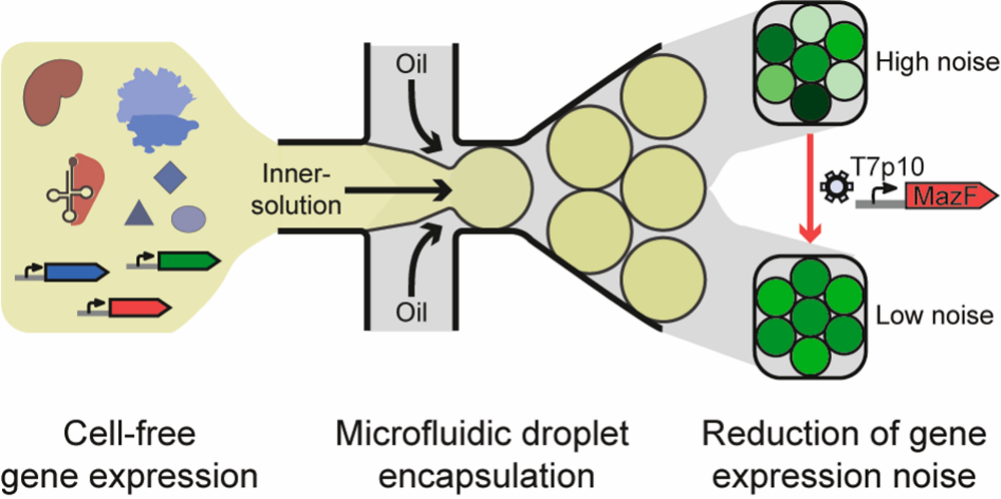

Biochemical reactions that involve small numbers of molecules are accompanied by a degree of inherent randomness that results in noisy reaction outcomes. In synthetic biology, the ability to minimize noise particularly during the reconstitution of future synthetic protocells is an outstanding challenge to secure robust and reproducible behavior. Here we show that by encapsulation of a bacterial cell-free gene expression system in water-in-oil droplets, *in vitro*-synthesized MazF reduces cell-free gene expression noise >2-fold. With stochastic simulations we identify that this noise minimization acts through both increased degradation and the autoregulatory feedback of MazF. Specifically, we find that the expression of MazF enhances the degradation rate of mRNA up to 18-fold in a sequence-dependent manner. This sequence specificity of MazF would allow targeted noise control, making it ideal to integrate into synthetic gene networks. Therefore, including MazF production in synthetic biology can significantly minimize gene expression noise, impacting future design principles of more complex cell-free gene circuits.

## Introduction

Approaches to building synthetic or minimal cells have gained increased traction in recent years. Top-down methods provide a vital platform to identify key cellular processes required for minimal cells to function.^1,2^ Conversely, elegant bottom-up studies have reconstituted bacterial processes such as membrane synthesis^4^ and division machinery,^3,4^ signaling pathways,^5^ DNA replication,^6,7^ and cell-free gene expression systems.^8−10^ To ultimately combine these individual modules into a functional synthetic cell, coordinated gene expression is crucial. Much work focuses on designing and characterizing synthetic gene networks in batch systems^11−13^ or more recently using microfluidic flow reactors.^14−16^ However, the inherent stochastic nature of biochemical reactions generates a high degree of variation (*i.e.*, “noise”) in reaction outcome when performed in a confined space.^17−19^ This noise has been shown to be detrimental for reliable function of synthetic circuits in bacteria^20^ and will therefore likely impede the robust and reproducible construction of synthetic cells with ultimate therapeutic and diagnostic applications.

*In vivo*, cells ensure reliable and robust function by implementing various noise minimization strategies, which range from complex architectures^21^ to more simple solutions.^22^ Generally, cellular noise minimization can be achieved by a high transcription rate followed by low nuclear export, high cytoplasmic mRNA degradation, or low translation rate.^22−24^ Other efficient strategies involve negative autoregulatory feedback motifs, which are often post-transcriptional in nature.^25,26^ While both strain and gene sequence engineering have been applied to reduce noise in microbial cell factories,^20^ studies aimed at minimizing noise in *in vitro* systems have been lacking.

**Figure 1:**
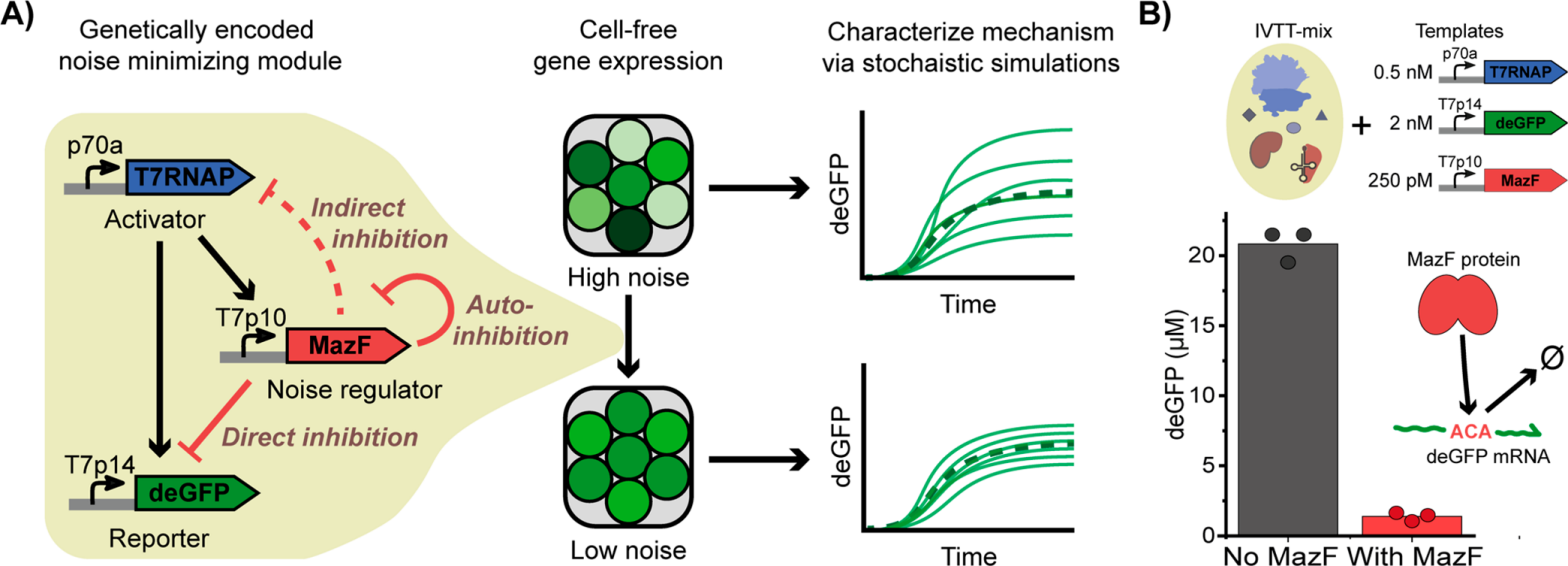
**Overview of the effects of T7p10-MazF on cell-free gene expression.** (A) Genetic composition of the noise-minimizing module applied in cell-free gene expression system (left). Adding the MazF-expressing template reduces the deGFP expression noise (middle). To understand the mechanism of noise modulation, stochastic simulations were performed (right). (B) A cell-free gene expression reaction is composed of an *in vitro* transcription–translation (IVTT) mix and DNA templates (top). deGFP expression yields of 2 nM T7p14-deGFP in the absence and presence of 250 pM T7p10-MazF (bottom). The expressed MazF protein reduces deGFP expression by promoting mRNA degradation in an ACA-sequence-specific manner (bottom right). Each point is the deGFP yield of one replicate.

Therefore, we set out to develop and characterize an *in vitro* noise minimization module (Figure 1A). To make it easily implementable, several design principles need to be taken into consideration. First, the module must be genetically encoded and consist of a minimal number of genes. Second, the DNA templates should be expressible in an *Escherichia coli*-based cell-free expression system, as this is generally used in the field.^27^ Third, the system should be able to modulate the gene expression noise of a target gene *via* one of the above-mentioned mechanisms. One gene that satisfies all these requirements is *mazF*, which is part of the mazEF toxin antitoxin system found endogenously in *E. coli*. The MazF protein is a sequence-specific ribonuclease that acts preferentially on ACA-sequence-containing single-stranded RNA, resulting in inhibition of translation through site-specific cleavage (Figure 1B).^28^ Finally, MazF has been implicated in gene expression noise modulation *in vivo*(29) and is compatible with cell-free gene expression.^30^ However, employing MazF to minimize noise in cell-free gene expression has not been attempted and could offer a powerful tool to reproducibly control cell-free systems. This motif could therefore be easily integrated into larger cell-free circuits to control the noise levels.

Here we incorporate the *mazF* gene into a cell-free genetic network, with the goal of minimizing gene expression noise. To this end, we first show successful synthesis of the MazF protein from a DNA template in our cell-free gene expression system and characterize the effect on the expression of a reporter green fluorescent protein (deGFP). Moreover, by exploiting droplet-based microfluidics, we find that gene expression noise is reduced >2-fold by MazF. Finally, by employing stochastic modeling we identify that both increased degradation and the autoregulatory feedback of MazF contribute to the observed noise reduction. Taken together, these results show that *mazF* can be used as a noise-minimizing module that could provide an easily implementable and tunable noise minimization strategy for future synthetic cells and cell-free biosensors.

## Results and Discussion

To test the potential noise-reducing effect of the *mazF* gene *in vitro*, we incorporated it into a genetic network with a feed-forward loop topology. Feed-forward loops are commonly found *in vivo*(31) and are widely applied to construct synthetic networks.^15,32,33^ To build this network, three linearized DNA templates were constructed (see Methods for more details): a T7 RNA polymerase (T7RNAP)-expressing sequence driven by a strong endogenous promoter (p70a-T7RNAP), a deGFP reporter sequence controlled by an exogenous T7 promoter (T7p14-deGFP), and a MazF-expressing sequence also controlled by a T7 promoter (T7p10-MazF). The T7RNAP and deGFP templates were previously reported,^8^ while T7p10-MazF was constructed in this work (Table S1). These templates were expressed in an *E. coli*-based (BL21 Star) cell-free expression system, and reaction progression was followed by measuring deGFP fluorescence. The deGFP synthesis rates were controlled by varying the T7p14-deGFP and T7p10-MazF template concentrations, which respectively modulate the transcription and RNA degradation rates. The template concentration of p70a-T7RNAP was kept constant throughout this work. Expression of 2 nM T7p14-deGFP in the presence of only 250 pM T7p10-MazF template resulted in a 15-fold drop in protein yield (Figure 1B) as well as a 5-fold decrease in overall protein expression rates (Figure S2). These results illustrate that MazF can be synthesized in our cell-free expression system and inhibits deGFP expression.

**Figure 2:**
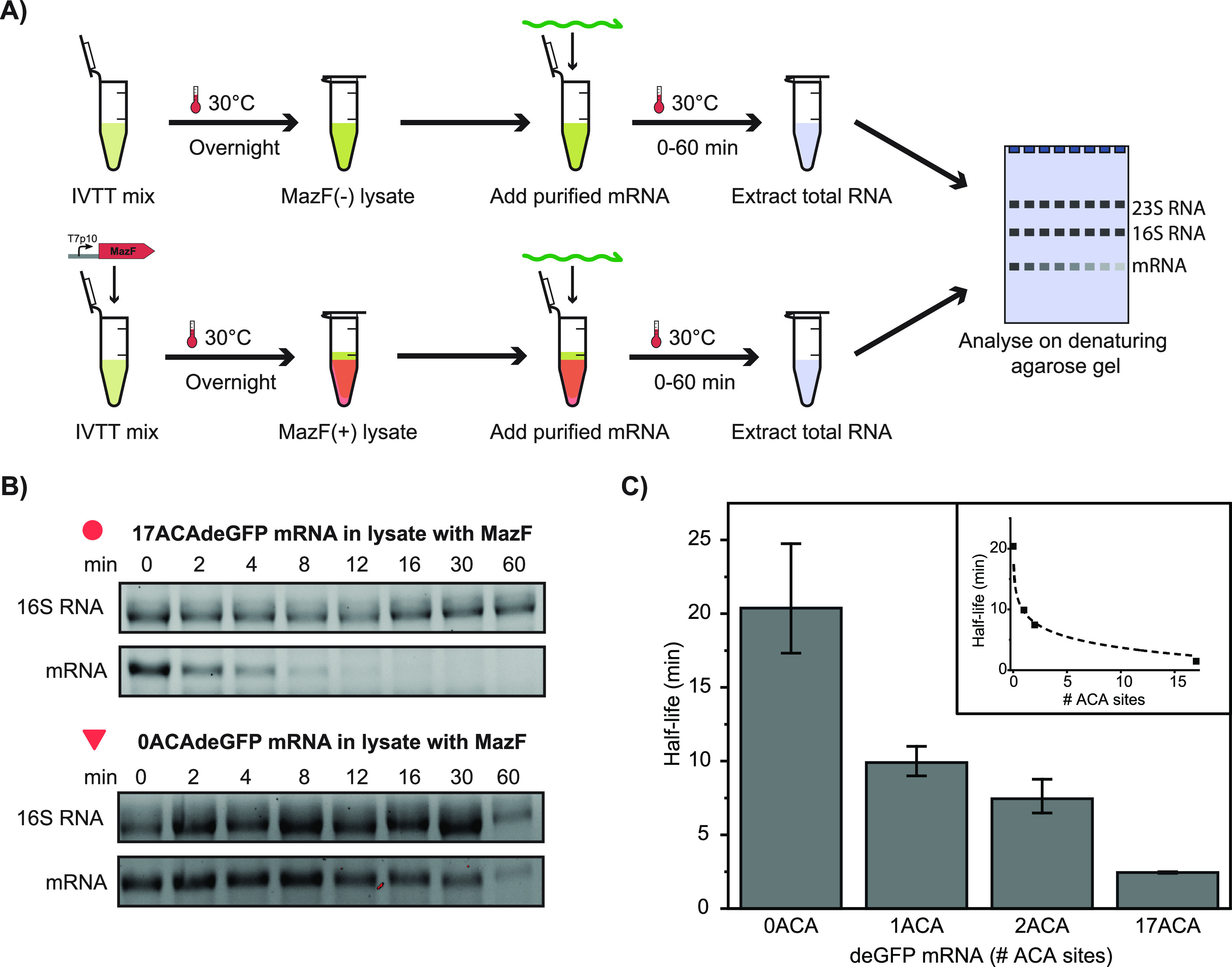
**Effect of overexpressed MazF protein on mRNA degradation.** (A) Schematic overview of the experimental workflow. (B) Denaturing agarose gel images of mRNA incubated in IVTT mixtures. Purified wild-type 17ACAdeGFP mRNA (top) or recoded 0ACAdeGFP mRNA (bottom). (C) Half-life of deGFP mRNA with 0, 1, 2, or 17 ACA sites in IVTT expression mixture containing overexpressed MazF. The bar plot displays the half-life values obtained from fitting an exponential decay function to the band intensities of the denaturing agarose gels (Figures 2B and S3), and the error bars represent the standard error of the fit. The inset shows the half-life of each mRNA versus the number of ACA sites in each mRNA (dashed line: half-life = 9.6 – 2.6 ln(# ACA sites)).

To confirm that this inhibition is caused by ACA-site-dependent mRNA degradation, we built deGFP-expressing constructs with zero, one, or two ACA sites. The convenience of the ACA-target sequence is that it is short enough to allow for mutations of the DNA sequence with retention of the amino acid composition of the protein product (Table S2). Next, to quantify the rates at which MazF degrades mRNA in the cell-free gene expression system, we performed an mRNA decay assay (Figure 2A). In short, after an overnight cell-free expression of 125 pM T7p10-MazF, 4 μM deGFP mRNA was introduced, and levels of deGFP mRNA were determined over time by gel electrophoresis (Figure S3). The effects of MazF on wild-type 17ACAdeGFP mRNA and on the recoded deGFP mRNA with zero, one, and two ACA sites were quantified over time by using the 16S rRNA band as an internal reference.^34^ The experiments yielded half-lives of 2.4 min for 17ACAdeGFP mRNA and 20 min for 0ACAdeGFP mRNA in the presence of MazF (Figure 2B,C,), *i.e.*, an 8-fold increase in degradation rate. Furthermore, we observed a clear dependence of the half-life on the number of ACA sites in deGFP mRNA (Figure 2C inset and Figure S3). As expected, MazF also degrades both its own mRNA and T7 RNAP mRNA since both these transcripts also contain ACA sites (Figures S4 and S5). While the degradation rates of 17ACAdeGFP mRNA and 0ACAdeGFP mRNA in the absence of MazF are not significantly different, the presence of MazF increases the degradation rates of 0ACA and 17ACAdeGFP mRNA 3- and 18-fold, respectively (Table S3). The effect of MazF on 0ACAdeGFP mRNA was unexpected but may be caused by aspecific cleavage due to a high concentration of expressed MazF combined with a lack of ACA-site-containing RNAs. Nevertheless, these data show that MazF has a strong preference for ACA-containing mRNA.

**Figure 3:**
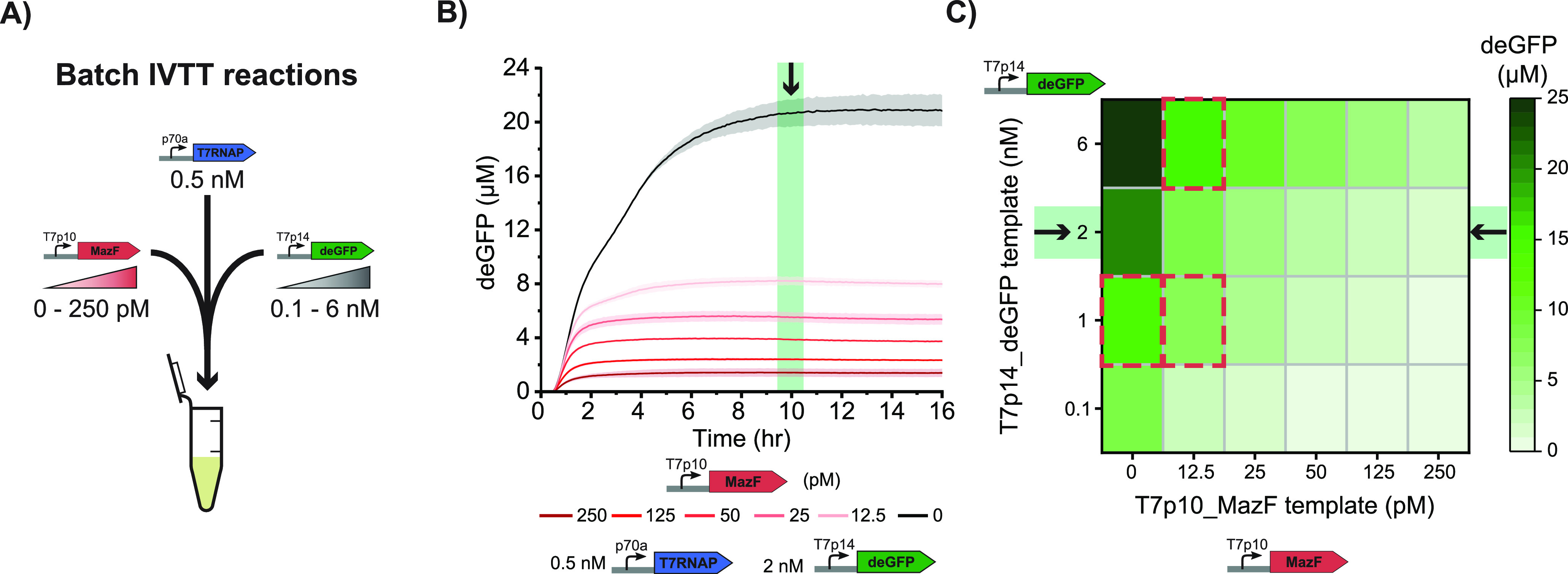
**Batch expression of a range of T7p14-deGFP and T7p10-MazF template combinations.** (A) Schematic overview of experimentally tested conditions. (B) Effect of increasing concentrations of the T7p10-MazF template on deGFP synthesis. The yields after 10 h of expression (highlighted in green) were used in (C). (C) deGFP yields after 10 h of expression for a range of T7p10-MazF and T7p14-deGFP concentrations. The red squares represent the three template combinations used for the droplet experiments. All time courses are shown in Figure S7.

Next, to characterize the effect of the T7p10-MazF template on the expression of T7p14-deGFP, a set of 24 different template combinations were tested (Figure 3A). Since 250 pM T7p10-MazF template almost completely inhibited the expression of deGFP (Figure 1B), first a range of lower T7p10-MazF template concentrations was coexpressed with 2 nM T7p14-deGFP (Figure 3B). Even at the lowest tested concentration of 12.5 pM T7p10-MazF, a significant reduction of deGFP expression was observed. Increasing the concentration of T7p10-MazF template from this point resulted in a further reduction of deGFP yields. To confirm that MazF acts on other ACA-containing reporter proteins, we tested the same range on T7p14-mCherry expression and observed a similar trend (Figure S6). Next, the same T7p10-MazF template range was tested with three additional concentrations of T7p14-deGFP template (0.1, 1, and 6 nM) (Figure S7). The results confirmed that both deGFP production yields and rates can be independently modulated by either T7p10-MazF or T7p14-deGFP template concentrations (Figures 3C and S8). Therefore, both the T7p10-MazF and T7p14-deGFP templates provide independent handles to tune protein levels in the cell-free gene expression system.

**Figure 4:**
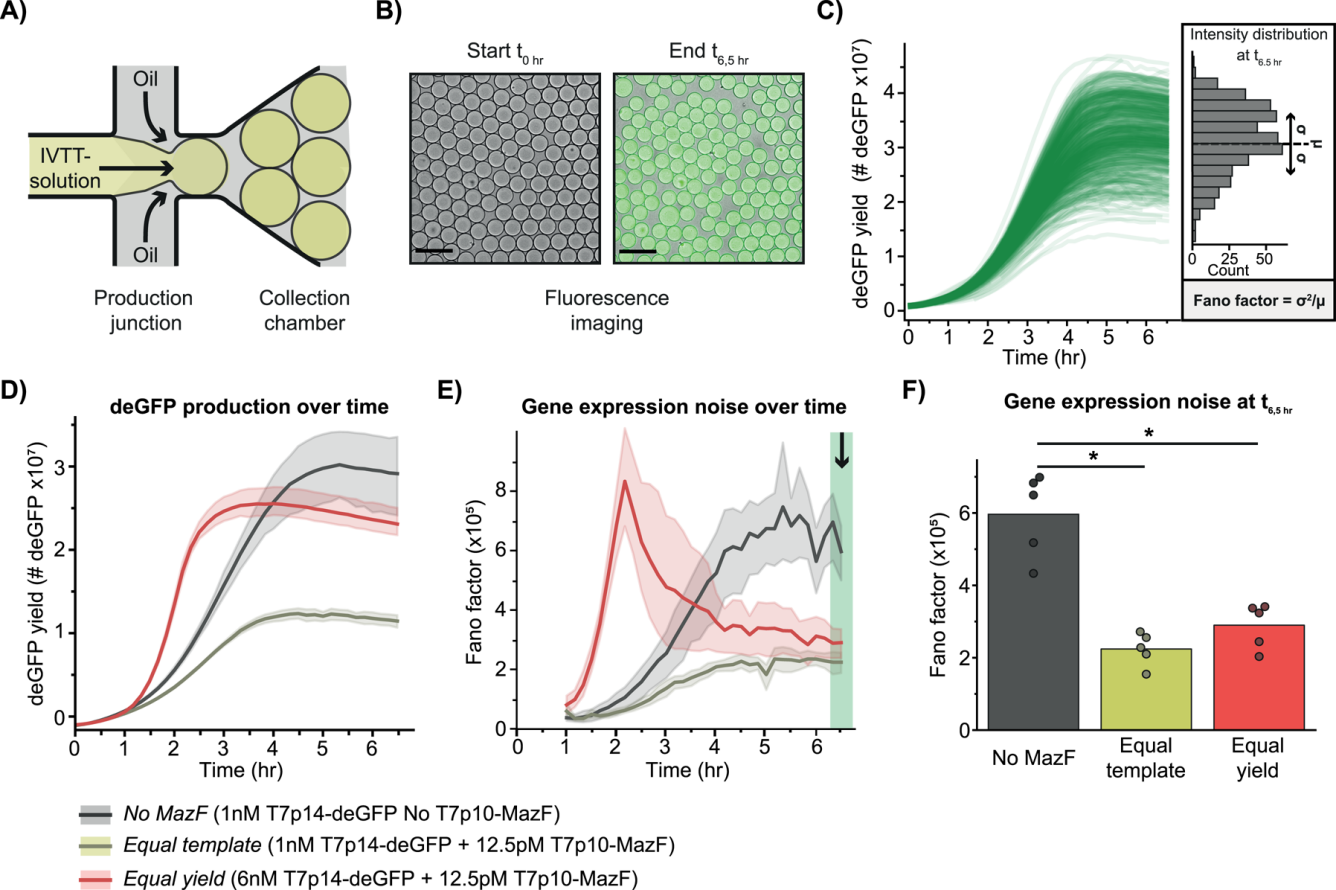
**Implementing the noise-minimizing module in picoliter droplets.** (A) Schematic overview of the microfluidic droplet production device. (B) Representative microscope images of the droplets immediately after production (left) and at the end of the experiment (right). Scale bars = 100 μm. (C) Single droplet trajectories of deGFP yield for the expression from the 1 nM T7p14-deGFP template. The histogram (right) represents the distribution of droplet intensities after 6.5 h. Mean (μ) and standard deviation (σ) are used to calculate the gene expression noise (Fano factor = σ^2^/μ). (D) deGFP yield for three tested conditions, with five positions per condition. (E) Average gene expression noise (Fano factor, see (C)) for the three tested conditions, with five positions per condition. The noise values after 6.5 h (highlighted in green) were used in (F). (F) Fano factor for the three tested conditions after 6.5 h. Each point is the Fano factor of a single position.

To study the effect of the MazF template on gene expression noise, a set of gene expression reactions were performed in droplets with a diameter of ∼30 μm. For these droplet experiments, we selected three template conditions: (i) 1 nM T7p14-deGFP with no T7p10-MazF (*i.e.*, *No MazF*); (ii) 1 nM T7p14-deGFP with 12.5 pM T7p10-MazF (*i.e.*, *Equal template*: equal deGFP template concentration as (i)); and (iii) 6 nM T7p14-deGFP with 12.5 pM T7p10-MazF (*i.e.*, *Equal yield*: comparable deGFP yield as (i)) (Figure 3C, red squares). Next, a PDMS-based microfluidic device was implemented to create a monodisperse population of water-in-oil droplets. The inner solution was composed of the complete gene expression mixture and the outer solution of 5% 008-FluoroSurfactant in FC40 oil (Ran Biotechnologies) (Figure 4A). After filling the collection chamber with droplets, the droplet production was stopped, and deGFP expression was followed over time (Figure 4B). To exclude the influence of differences in droplet sizes on the expression data, only droplets with a certain radius were considered. Tracking individual droplets over time demonstrated that all droplets exhibit similar expression dynamics but varied in the expression yields (Figures 4C, S9, and S10). Next, the average deGFP yield (Figures 4D and S11A–C) was quantified per position (*i.e.*, technical replicate) over time for all three template conditions (i–iii), and position-based extrinsic noise was filtered out (Figures S12 and S13). Although the onset of deGFP expression is more delayed in droplets compared to bulk expression, the deGFP expression curve reaches a plateau after ∼6 h, comparable to bulk experiments (Figure 4D compared to Figure 3B). Furthermore, the relative differences in deGFP yield are slightly different in droplets and in bulk, especially for the *No MazF* condition compared to the *Equal yield* condition (Figure S14). Consequently, to minimize the influence of different deGFP yields, deGFP noise was quantified as the Fano factor, given by σ^2^/μ (Figure S15).^23^ Strikingly, expressing MazF in these picoliter-sized droplets significantly reduces the noise in deGFP expression (Figures 4E,F and S11D–F). Interestingly, for the *Equal yield* condition (*i.e.*, condition (iii): 6 nM T7p14-deGFP with 12.5 pM T7p10-MazF) there is an initial stark increase in noise, followed by a decrease (Figure 4E, red). This increase in noise coincides with the earlier increase in deGFP expression (Figure 4D, red), and the decrease in noise coincides with the deGFP expression curve starting to plateau. Therefore, the observed overshoot in noise is likely caused by a faster initial increase in expression due to the higher T7p14-deGFP template concentration, which initially outcompetes the amount of MazF present. Once MazF is dominant, the mRNA is rapidly degraded and the noise in gene expression likely reduced. Conversely, at lower deGFP template conditions, both the start of expression and the plateau occur later, and no overshoot in gene expression noise is observed (Figure 4D,E, yellow). Here, due to lower concentrations of T7p14-deGFP template, it is possible that MazF outcompetes mRNA production from a much earlier time point, preventing an initial stark increase in noise. Importantly, in the presence of *mazF* template, deGFP expression noise is reduced >2-fold after 6.5 h irrespective of the deGFP template concentration (Figure 4F). Lastly, we confirmed that this noise reduction does not occur for the 0ACAdeGFP template (Figure S16).

Finally, to gain a quantitative understanding of our system, we developed a stochastic model to describe our experimental observations from a set of coarse-grained differential equations:
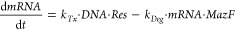
1
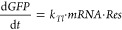
2where *k*_*Tx*_, *k*_*Tl*_, and *k*_*Deg*_ are the deGFP transcription, translation, and mRNA degradation rate constants respectively, *DNA* is the template concentration, and *Res* is the amount of finite and shared resources of transcription and translation machinery, which depletes over time (see Methods, eqs 5 and 6).^35^ Lastly, *MazF* is the amount of MazF protein present in the system, which is simulated in a similar way to the deGFP production (see Methods, eqs 3 and 4).

**Figure 5:**
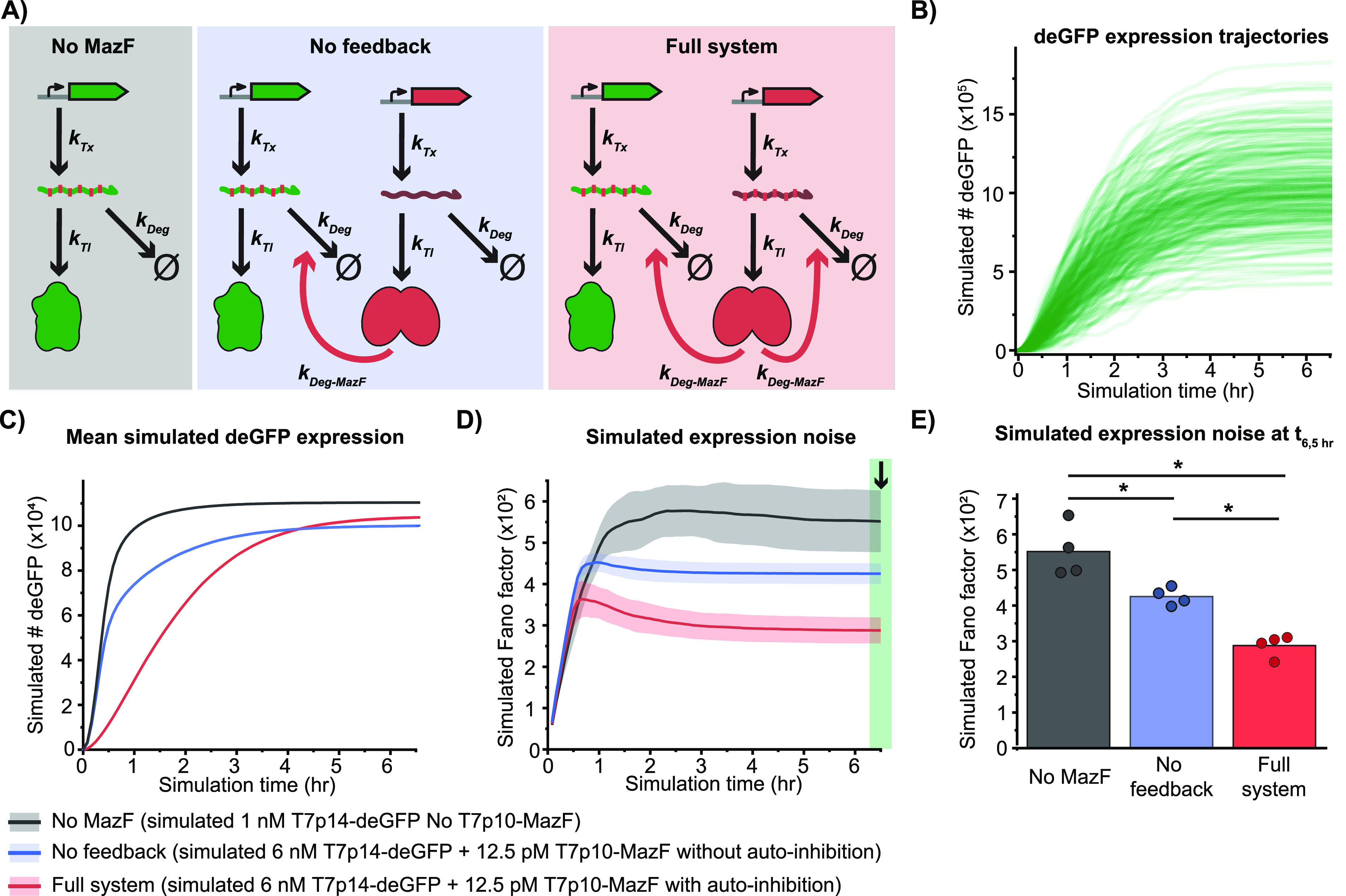
**Stochastic modeling of the effect of MazF on gene expression noise.** (A) Schematic representation of the three analyzed models: *No MazF* (only T7p14-deGFP template present (gray)), *No feedback* (MazF acts on the degradation of deGFP mRNA but not its own (blue)), and *Full system* (MazF acts on the degradation of both the deGFP mRNA and its own mRNA (red)). (B) Single trajectories of the simulated deGFP production for the *No MazF* model; 250 traces were randomly selected from the complete set of 1000 traces. (C) Mean deGFP production of all trajectories over time for all three models. (D) Average Fano factor of the deGFP yield over time for all three models. The noise values after 6.5 h (highlighted in green) were used in (E). (E) Analysis of the Fano factor at the 6.5 h simulated time point. Each point is the Fano factor of a subsampled population of 250 simulated droplets.

To explore the influences of MazF on gene expression noise, we used Gillespie’s direct method^36^ to perform stochastic numeric simulations of three different conditions: (i) no *mazF* template and 1 nM deGFP template (Figure 5A, gray—*No MazF*), (ii) 6 nM deGFP template in addition to *mazF* template which lacks any negative feedback on itself (Figure 5A, blue—*No feedback*), and (iii) 6 nM deGFP template and *mazF* template including the autoinhibition of MazF (Figure 5A, red—*Full system*). We simulated gene expression in 1000 individual droplets per condition (Figures 5B and S17) and quantified both mean deGFP expression (Figure 5C) and the Fano factor (Figure 5D). To reduce computational time, we excluded the expression of T7RNAP from the model and implemented lower *k*_*Tx*_ and *k*_*Tl*_ rates, resulting in lower deGFP expression values than observed experimentally. Although these simulated gene expression and corresponding noise values are lower than the experimental values, the simulations confirm that MazF synthesis significantly reduces gene expression noise (Figure 5E). Notably, the experimentally observed overshoot is less prominent *in silico*, indicating that the model does not capture all of the subtleties of the cell-free gene expression reactions. However, in line with our hypothesis, the overshoot coincides with a stark decrease in the level of deGFP mRNA in the presence of MazF *in silico* (Figure S17). Furthermore, we find that the noise reduction abilities of MazF are due to both the increased degradation of MazF on deGFP mRNA (Figure 5E, blue—*No feedback*) as well as the autoinhibition of MazF (Figure 5E, red—*Full system*). In other words, MazF reduces noise by acting as a quencher of the *in vitro* transcription–translation (IVTT) reaction through increased mRNA degradation as well as by autoregulating its own abundance through the autoregulatory feedback. Furthermore, we find that the noise reduction ability of MazF holds for a range of transcription and translation rates (Figure S18A, blue). Interestingly, when the rates are reduced to such an extent that a considerable number of simulated droplets contain no MazF (Figure S18B,C), the effect on gene expression noise could be reversed (Figure S18A, red). Lastly, we find *in silico* that MazF successfully reduces the noise of the response of a transcriptional riboswitch to an analyte (Figure S19). Collectively, these data show that coexpression of MazF in droplets provides an independent handle to control gene expression noise.

## Conclusion

We successfully constructed a noise-minimizing genetic module by incorporating the *mazF* gene into a feed-forward loop topology. Our results show that MazF can be synthesized in a cell-free gene expression system and inhibits expression of both the deGFP and mCherry reporter genes by targeting mRNA containing ACA sites. Specifically, the mRNA degradation rate of regular deGFP mRNA is increased ∼18-fold in the presence of MazF. We have established that both the T7p10-MazF and T7p14-deGFP templates and the number of ACA sites provide independent handles to tune protein levels in the cell-free gene expression system. By exploiting droplet-based microfluidics, we have demonstrated that MazF synthesis reduces deGFP expression noise >2-fold. Finally, we have confirmed this noise reduction *in silico* and identified both increased mRNA degradation and autoinhibition of MazF as contributing factors to the observed noise minimization.

Provided that the experimental system allows for control over the added DNA constructs, this technique would enable the noise control of specific targets in a large range of genetic networks. The tunability of the noise reduction module depends on template stoichiometry, the number of ACA sites of the template, and the transcription and translation rates of the cell-free gene expression system used. The most straightforward way in which MazF-mediated noise reduction can be implemented is through template stoichiometry. Since most genes naturally contain at least one ACA site, this noise minimization would take effect without requiring recoding of templates. However, recoding genes to create 0ACA variants, while more time-consuming, would allow for more selective noise reduction. Moreover, MazF has previously been shown to be sensitive to *N*^6^-methyladenosine,^37^ which might provide a separate handle to tune the effect of MazF on gene expression noise. Importantly, the short target sequence of MazF permits the alteration of ACA sites in a gene of interest without disrupting its function due to degeneration in the genetic code. Simulations predict that the noise reduction of MazF can be further tuned by altering transcription and translation rates, and this module might even be able to generate noise amplification.

Lastly, the sequence specificity of MazF is rare among naturally occurring ribonucleases, making it ideal to integrate into synthetic gene networks because it allows targeted noise control. Interestingly, MazF variants found in other bacteria have different sequence specificities, highlighting the potential to construct variations on the module in the future.^38^

## Methods

### *In Vitro* Transcription–Translation

An IVTT reaction is composed of three main elements: lysate, feeding buffer, and DNA templates. The lysate and feeding buffer were previously described.^15^ In this work, however, *E. coli* BL21 Star (transformed with the pRARE plasmid as described in ref (32)) was used for the preparation of lysate, which has reduced intrinsic RNA degradation levels.

For the IVTT reaction, a master mix was prepared from lysate (∼10 mg/mL total protein content), feeding buffer (1 mM DTT, 1.5 mM each amino acid, 50 mM HEPES, 1.5 mM ATP and GTP, 0.9 mM CTP and UTP, 0.2 mg/mL tRNA, 0.26 mM CoA, 0.33 mM NAD, 0.75 mM cAMP, 0.068 mM folinic acid, 1 mM spermidine, and 30 mM 3-PGA), magnesium glutamate (6 mM), potassium glutamate (40 mM), maltose (75 mM), PEG-8000 (2%), and GamS (∼2 μM purified following a previously published protocol^39^). For the droplet experiments, 1.33 units/mL inorganic pyrophosphatase (NEB) was added. All other components were purchased from Sigma-Aldrich.

For batch IVTT reactions, the master mix was added to the linearized DNA templates in reactions of 10 μL. From this mixture, 9.5 μL was loaded into a flat-bottom nonbinding 384-well plate (Greigner Bio-one) and covered with a coverslip. Fluorescence was measured at 30 °C on an Infinite 200 PRO or Spark 10 M plate reader (both Tecan). The raw fluorescence units were converted to μM using a linear calibration curve of titrated fluorescent protein (Figure S20A–C).

### mRNA Degradation Assay

An IVTT reaction mixture (150 μL) was prepared as described above either with only 0.5 nM p70a-T7RNAP template (MazF(−)) or with an additional 125 pM T7p10-MazF (MazF(+)). This reaction mixture was incubated at 30 °C overnight to allow the expression to plateau. Next, 12.5 μL of the reaction mixture was aliquoted and mixed with an equal volume of purified mRNA (1 μg/μL f.c., corresponding to 4 μM for deGFP mRNA). This mixture was incubated for 0–60 min at 30 °C, after which the reaction was quenched by the addition of 100 μL of phenol/chloroform/isoamyl alcohol (125:24:1, Sigma-Aldrich) and the mixture was stored on ice until extraction.

To extract the RNA from the reaction mixture, 75 μL of UltraPure water (Invitrogen) was added to increase the volume of the aqueous phase. After mixing and centrifugation, the aqueous phase was removed, and 1 μL of glycogen (15 mg/mL stock, Sigma-Aldrich), 0.1 volume of sodium acetate (3 M stock, Fluka), and 2.5 volumes of ice-cold ethanol (75%, Merck) were added. After incubation for >30 min at −20 °C, the precipitated RNA was collected by centrifugation and resuspended in 25 μL of UltraPure water.

The samples were analyzed on a denaturing agarose gel. The gel was made by dissolving 0.5% agarose (Fisher Scientific) in 0.1 volume of 10× MOPS-buffer (MOPS (200 mM, Sigma-Aldrich), sodium acetate (50 mM), Na_2_EDTA (10 mM, Sigma-Aldrich) to pH 7.0) with 0.18 volume of formaldehyde (37–41%, Fisher Scientific). The extracted RNA (5 μL) was mixed with 16 μL of sample buffer (5:2:1 formamide (Fisher Scientific)/formaldehyde/10× MOPS), 1 μL of bromophenol blue (50% stock, Merck), and 1 μL of ethidium bromide (5 mg/mL stock, Sigma-Aldrich). After the samples were denatured for 10 min at 75 °C, the gel was run at 100 V for 1 h and imaged on a Gel Doc XR+ imager (Bio-Rad). The images were analyzed using Image Lab 6.1 (Bio-Rad).

### Microfluidic Device Construction and Encapsulating IVTT Reactions

The PDMS-based microfluidic droplet devices were made as previously described^18^ with some modifications. The wafers used for the production of the devices had an average height of 20 μm. To create the hydrophobic coating, a 2% silane solution (1*H*,1*H*,2*H*,2*H*-perfluorooctyltrichlorosilane (97%, Thermo Fisher Scientific) in Fluorinert FC-40 oil (ChemCruz)) was flushed through the device before it was baked at 100 °C for at least 3 h.

To generate droplets, syringe pumps were connected using PTFE tubing (0.56 mm i.d., 1.07 mm o.d., VWR). The outer solution (5% 008-FluoroSurfactant in FC-40 oil (Ran Biotechnologies)) was injected with a flow rate of 15–70 μL/h. For the inner solution, an IVTT mixture without template (see above) was incubated for 30 min at 37 °C. After addition of the linearized templates, the solution was injected into the device at a flow rate of 15–40 μL/h. The droplets were imaged every 10 min on an inverted microscope (Olympus IX81) equipped with a motorized stage (Prior, Optiscan II) using a 20× objective. Fluorescence images were taken with a sensitive electromultiplying charge-coupled device camera (iXon, Andor) using illumination from a mercury lamp (100 ms exposure).

### Stochastic Simulations

Stochastic cell-free gene expression in 1000 droplets was simulated using Gillespie’s direct method algorithm.^36^ The theoretical model describes deGFP transcription, translation, and mRNA degradation (eqs 1 and 2) as well as MazF transcription, translation, and mRNA degradation:
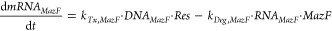
3
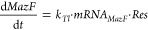
4

In order to exclude any effect on gene expression noise that might be caused by resource competition,^40^ depletion of resources was modeled to be independent of DNA, mRNA, or protein abundance.^41^ Instead, resource depletion was made dependent on a depletion factor (*DepF*) that depends on translation:
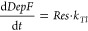
5and an additional depletion rate constant *k*_*dep*_.^35^
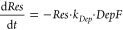
6

All rate constants used are reported in Table S5. For the riboswitch model, the transcription rate was made dependent on the concentration of an analyte. Two variants were constructed in which this dependence was either linear or sigmodal (Figure S19B).

### Statistical Tests

For all statistical tests, a two-sided Student’s *t* test was employed, and the significance level set to *P* < 0.05.

## Supporting Information Available

Supplementary methods (preparation of DNA templates, *in vitro* transcription, and image analysis workflow), Figures S1–S20 (kinetics of batch expressions, denaturing gel images, droplet trajectories and droplet characteristics, additional droplet experiments, trajectories for all components in the stochastic simulations, analysis of variations on the model, and calibration curves for fluorescent proteins), and Tables S1–S5 (DNA sequences, fitting parameters for RNA degradation, and input parameters for the simulations) (PDF)
