# Supplementary material for: Noise Minimization in Cell-Free Gene Expression
Source: ACS Synth Biol. 2023 Jul 21;12(8):2217–25. doi: 10.1021/acssynbio.3c00174 (PMC10443034; doi:10.1021/acssynbio.3c00174)
Supplement: Supplementary file 1 — sb3c00174_si_001.pdf [file sb3c00174_si_001.pdf]

Supplementary Information
for

# Noise Minimization in Cell-Free Gene Expression

Mart W. Bartelds[1], Óscar García Blay[1], Pieter Verhagen[1], Elise J. Wubbolts[1], Bob van Sluijs[1], Hans A. Heus[1], Tom F. A. de Greef[1-5], Wilhelm T. S. Huck[1], Maike M. K. Hansen[1]*.

**The Supplementary Information includes:**

**Supplementary methods:**
Preparation of DNA templates
*In vitro* transcription of mRNA
Image analysis and droplet selection
Figure S1: Droplet segmentation and filtering

**Supplementary figures:**
Figure S2: Expression kinetics of deGFP in the presence and absence of MazF
Figure S3: Denaturing agarose gels of deGFP mRNA degradation experiments
Figure S4: Denaturing agarose gels of MazF mRNA degradation experiments
Figure S5: Batch IVTT data using purified T7RNAP enzyme
Figure S6: Batch IVTT data showing the effect of MazF on mCherry expression
Figure S7: Time courses of deGFP expression for conditions in Figure 3C
Figure S8: Heatmap representing the maximum expression rates for the conditions in Figure 3C
Figure S9: Single droplet deGFP trajectories for *Equal template* and *Equal yield* experiments
Figure S10: Analysis of droplet characteristics per position
Figure S11: Analysis of droplet intensities per position
Figure S12: Histograms showing the distribution of deGFP production yield per droplet after 6.5 hours
Figure S13: Analysis per position removes position-based noise
Figure S14: Comparison of relative production yields between batch and droplet experiments
Figure S15: Effect of total deGFP production on the Fano factor and $CV^2$
Figure S16: The effect of MazF on the expression noise of the recoded T7p14-0ACAdeGFP template
Figure S17: Overview of individual trajectories from stochastic simulations
Figure S18: Stochastic simulations of deGFP synthesis in presence and absence of MazF synthesis module for a range of transcription and translation rates
Figure S19: Stochastic simulations of deGFP synthesis with a transcriptional riboswitch module
Figure S20: Calibration curves for fluorescent proteins

**Supplementary tables:**
Table S1: Table of templates used in this work
Table S2: Table of gene sequences for the plasmids constructed in this work
Table S3: Exponential decay parameters of mRNA degradation experiments
Table S4: Origins of DNA fragments used for cloning
Table S5: Input parameters used for stochastic simulations.

**Preparation of DNA templates**

All linear templates used in this work were made using PCR. PCR's were carried out using in-house purified Phusion polymerase in 5x-HF-buffer (NewEngland Biotechnologies) according to the manufacturers protocol. Purified plasmid template was purchased from ArborBiosciences for the p70a-T7RNAP and T7p14-deGFP plasmids (Table S1).

The T7p10-MazF plasmid was constructed by incorporating the *mazF* gene (Table S2) into the pET23b vector (Novagen), using restriction-ligation cloning. The 0ACAdeGFP, T7p10-17ACAdeGFP, and mCherry plasmids were constructed using the GoldenGate assembly protocol. In short, linear DNA fragments (table S4) were assembled by restriction-ligation cloning using the Esp3I and T4-ligase enzymes (both NEB) in a one step-protocol. The resulting ligation mixture was then transformed into *E. coli* Top10F cells, from which the plasmid was isolated using the QIAprep Spin Miniprep kit (Qiagen). The 1 and 2 ACA site containing deGFP plasmids were made by performing site-directed mutagenesis on the T7p10-0ACAdeGFP plasmid. The plasmids were amplified in and isolated from *E. coli* Top10F cells as described above. The sequences were verified by Sanger Sequencing (BaseClear).

***In vitro* transcription of mRNA**

deGFP mRNA was produced by *in vitro* transcription of linearized T7p10-*n*ACAdeGFP (n= 0, 1, 2, or 17), or T7p10-MazF template. For this a reaction mixture consisting of 5-50 nM DNA template, 40 mM Tris-HCl (pH 8.1, Merck), 25 mM $MgCl_2$ (Merck), 5 mM DTT (Fisher Scientific), 1 mM spermidine (Merck), 4mM rNTPs (UTP,CTP and GTP purchased from Jena Biosciences, ATP from Applichem), 5mM GMP (Sigma) and 0.1 volume T7-RNA polymerase (purified in-house) was incubated for 2-4 hours at 37 °C. After completion, 50 mM $Na_2$-EDTA (pH 8.3, Sigma-Aldrich) was added to dissolve the salt precipitates. Then, the mixture was diluted 4-fold in sodium acetate solution (final concentration 375 mM, Fluka) and precipitated using isopropanol (Sigma-Aldrich), followed by a washing step with 75% ethanol (Merck). Finally, the dried pellet was resuspended into UltraPure water (Invitrogen). The quality of the mRNA was verified by gel-electrophoresis and its concentration was determined using the Qubit RNA HS kit (Novagen).

**Image analysis and droplet selection**

For each experiment the transmission and fluorescence images were stored in a single folder for each imaged position. To extract the information of individual droplets an image analysis pipeline was constructed in Python 3.8 (software foundation, Delaware US). In brief, the image analysis pipeline consists of four steps: i) droplet segmentation; ii) filtering out incorrectly segmented droplets using a supported vector machine; iii) tracking droplets over time; and iv) selecting droplets for analysis. The first three steps of this pipeline followed the methodology previously described.[1]

The segmentation was performed per timepoint on the transmission images. For this the contrast in the image was enhanced using Contrast Limited Adaptive Histogram Equalization after it was binarized using OTSU-thresholding (OpenCV v4.5.4.58).[2] After filling up small holes in the binary image, the objects were segmented using a watershed algorithm. The coordinates of the segmented objects were then used to cut out the objects from the fluorescence image (Figure S1A, colored boxes).

Since the differences in intensity value in the transmission channel are relatively small, some of the segmented objects were actually background. Furthermore, some of the objects showed clear defects in the fluorescence channel. Therefore, a support vector machine model (Scikit-learn v1.0.1)[3] was trained to filter out these segmentation events. To train the model 3432 randomly selected segments from images from all three experiments were manually divided into three categories: 0: incorrect segmentation events (578 items), 1: correctly segmented droplets (2567 items), 2: correctly segmented droplets, but with clear defects in fluorescence channel (287 items) (Figure S1B). The model was trained on 50% of this data set, after which we verified that it could categorize the other half of the data set with very high accuracy (Figure S1C).

After selecting only droplets that were sorted into to category 1, a droplet object was created in which the following information was stored: the coordinates of the centroid (x, y and timepoint), surface (sum of all pixels in segmented object), the droplet radius (calculated from the surface using $r = \sqrt{surface/\pi}$ ) and the droplet fluorescence (sum of the raw fluorescence intensities of all pixels in a circle with a radius 0.5 times the full droplet radius around the centroid (Figure S9A)).

Before exporting the data, we aimed to track individual droplets throughout the experiment. Therefore, a three-dimensional graph was constructed from all the coordinates of all correctly segmented droplets in all time points using cKDTree (Scipy-spatial v1.7.3.). This package was then used to connect centers that showed limited movement between subsequent timepoints and awarded each set of connected droplets with a unique index number. For all correctly segmented droplets this index number was appended to the droplet information, after which the data was exported to an excel file.

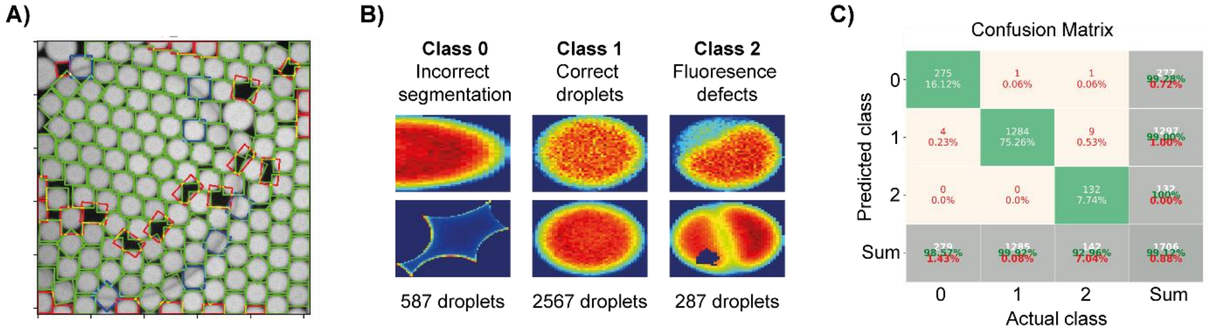


**Figure S1: Droplet segmentation and filtering. (A)** Example of segmented fluoresence image from the *No MazF* experiment. Each colored box represents one segmented object. The colors represent the category that was assigned to it (class 0: red, class 1: green and class 2: blue). **(B)** Examples of segmented objects assigned to each class. Class 0 contains incorrectly segmented objects, which are either background objects or incomplete droplets. Class 1 represent correctly segmented droplets and class 2 contain droplets that showed defects in the fluoresence channel. **(C)** Confusion matrix of the the categorization of the supported vector machine model for the test data. Of the droplets that were predicted to be class 1 only 1% was actually belonging to a different class.

After importing the droplet data using pandas (v1.0.1),[4] the droplet radius was converted from pixels to µm (100 µm = 127 pixels) and the fluorescence intensity to number of deGFP molecules (#deGFP). First, the background fluorescence (mean droplet intensity of the first time point) was subtracted from the mean droplet fluorescence. Then, the mean intensity was converted to the deGFP concentration using the calibration curve shown in Figure S20D.

Finally, the concentration was converted to number of molecules using the volume of the cylinder as shown in Figure S9A.

To select droplets for analysis, two filters were applied to the data set: a droplet-size based filter, and a position-based noise filter. The droplet-size based filter was applied to limit the effect of size variation on the final data. To account for droplet shrinkage over time a gradually decreasing size range was chosen (15% decrease in 400 minutes). For example, for the experiments in the main text only droplets were selected with a radius smaller than 18 µm – 18 * timepoint * 0.15/40 µm and larger than 16 µm – 16*timepoint* 0.15/40 µm. The position-based noise filter was applied to remove variation between the mean intensity of different microscope positions within the same experiment. To calculate the mean and noise statistics of each analyzed parameter, the droplets were grouped per microscopic position (i.e., field of view) and each of these positions was treated as an experimental replicate.

Data was plotted using either OriginPro (2020b v9.7.5.184) or using the Python packages using Matplotlib (v3.1.3) and Seaborn (v0.10.0).[5] For the single trajectory data another filter was applied based on the number of connected droplets. Only droplets that could be connected over 10 timepoints were shown in the graphs.

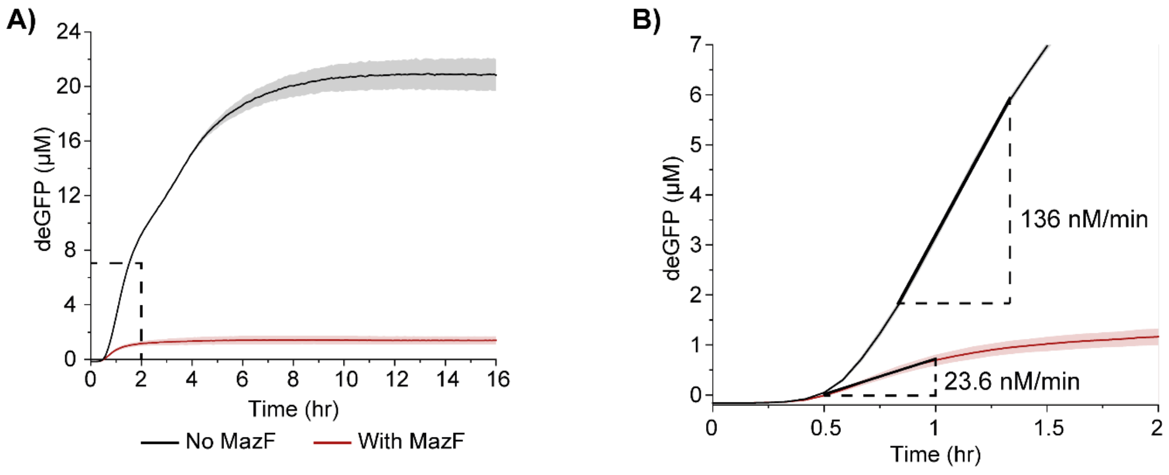


**Figure S2. Expression kinetics of deGFP in the presence and absence of MazF. (A)** Time course data of experiments shown in Figure 1B. 0.5 nM p70a-T7RNAP was co-expressed with 2 nM T7p14-deGFP template in the presence (red) or absence (black) of 250 pM T7p10-MazF template. **(B)** Zoom in on the start of the time courses (dashed box shown in A). The dotted lines indicate the 30-minute timeframe in which the maximum expression rate (written in the graph) was recorded.

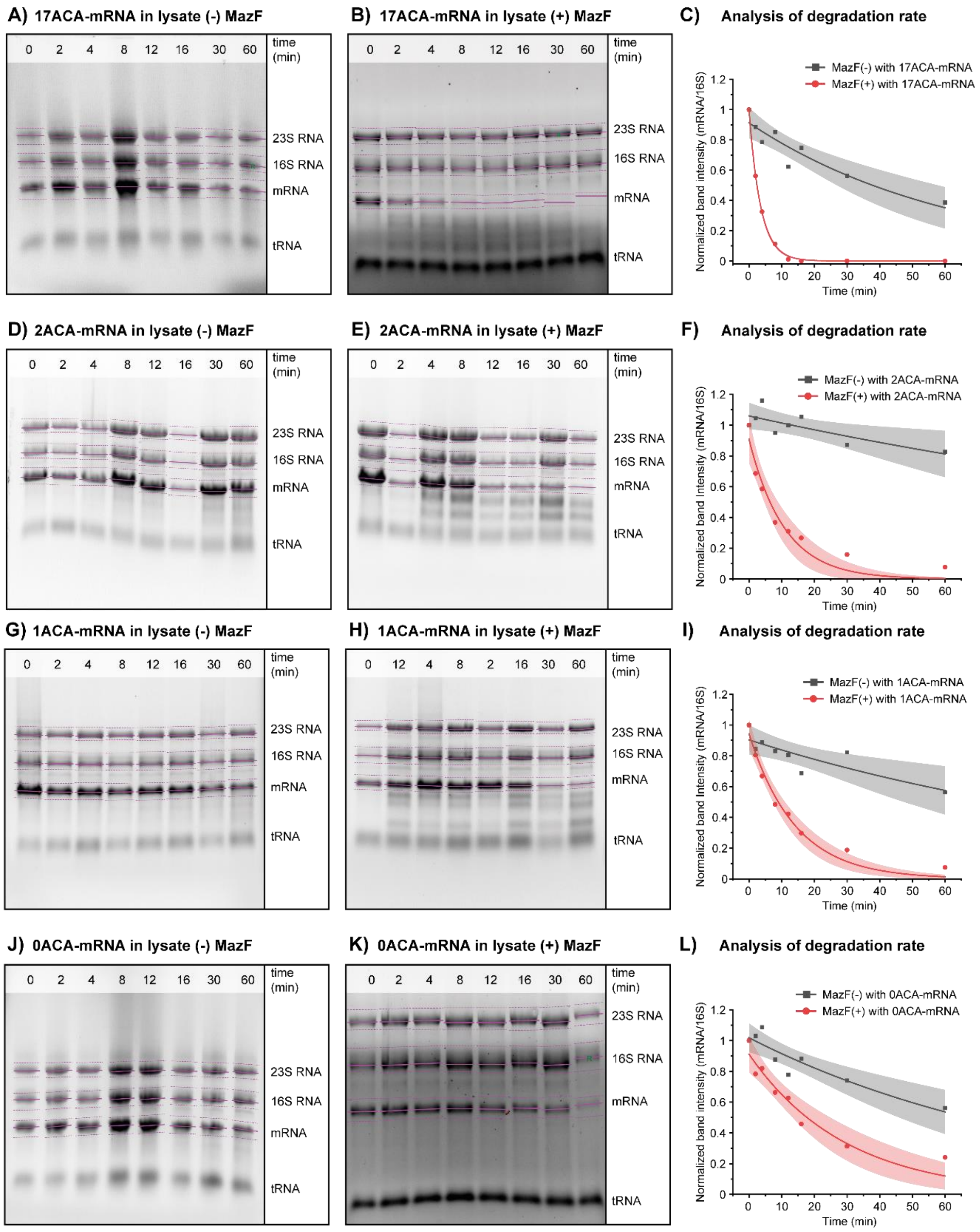


**Figure S3. Denaturing agarose gels of deGFP mRNA degradation experiments. (A,B,D,E,G,H,J,K)** Denaturing agarose gels for deGFP mRNA degradation experiments. The total RNA was isolated from the IVTT-mixture at the indicated times after addition of the purified mRNA. Besides the added mRNA, also the ribosomal RNA (23S and 16S) and tRNA bands were detected. Purple bands highlight selected areas for band intensity analysis shown in (C,F,I,L). **(C,F,I,L)** Decrease in mRNA abundance over time. The symbols represent the relative intensity of the mRNA band with respect to the 16S ribosomal RNA band. The mRNA was incubated in an IVTT expression mixture without MazF (black) or with overexpressed MazF (red). The lines through the points are exponential decay fits shown with a 95% confidence interval (shaded regions).

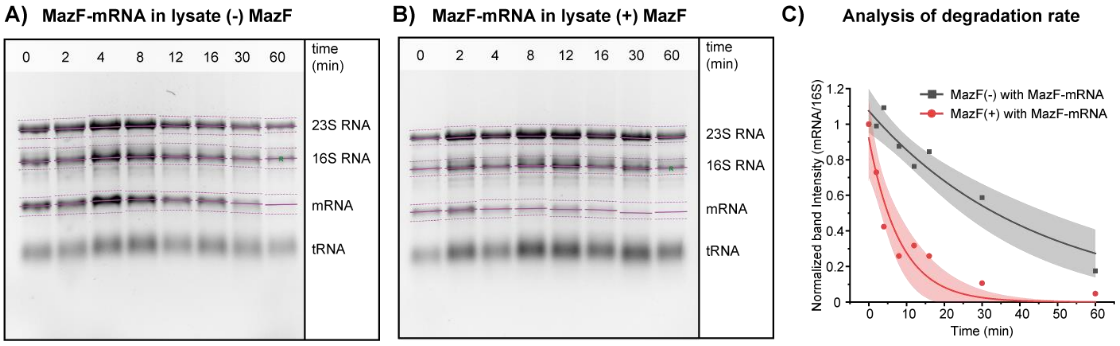


**Figure S4. Denaturing agarose gels of MazF mRNA degradation experiments. (A,B)** Denaturing agarose gels for MazF mRNA (11 ACA sites) degradation experiments. The total RNA was isolated from the IVTT-mixture at the indicated times after addition of the purified mRNA. Besides the added mRNA, also the ribosomal RNA (23S and 16S) and tRNA bands were detected. Purple bands highlight selected areas for band intensity analysis shown in (C). **(C)** Decrease in mRNA abundance over time. The symbols represent the relative intensity of the mRNA band with respect to the 16S ribosomal RNA band. The mRNA was incubated in an IVTT expression mixture without MazF (black) or with overexpressed MazF (red). The lines through the points are exponential decay fits shown with a 95% confidence interval (shaded regions).

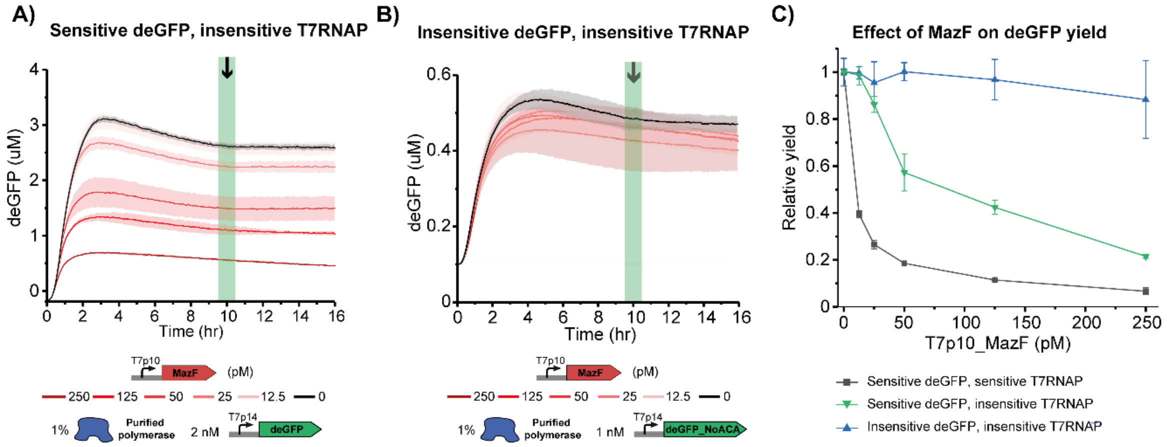


**Figure S5. Batch IVTT data using purified T7RNAP enzyme.** To show that MazF also targets the T7RNAP mRNA, two experiments were performed with purified T7RNAP enzyme (i.e. insensitive T7RNAP) instead of its template (i.e. sensitive T7RNAP). **(A, B)** Time courses for the deGFP expression from the T7p14-17ACAdeGFP template (A, sensitive deGFP) or T7p14-0ACAdeGFP template (B, insensitive deGFP). **(C)** Comparison of the effect of T7p10-MazF on the deGFP yields normalized to the no MazF condition for three experiments. Grey: sensitive deGFP with sensitive T7RNAP (corresponding to Figure 3B). Green: sensitive deGFP with insensitive T7RNAP (corresponding to A). Blue: insensitive deGFP with insensitive T7RNAP (corresponding to B). Removal of the effect of MazF on the T7RNAP (grey vs green) results in an increased deGFP expression yield, showing that T7RNAP is actively degraded in the presence of MazF. Removing the sensitivity of both deGFP and T7RNAP (blue) results in a system that is completely insensitive to MazF.

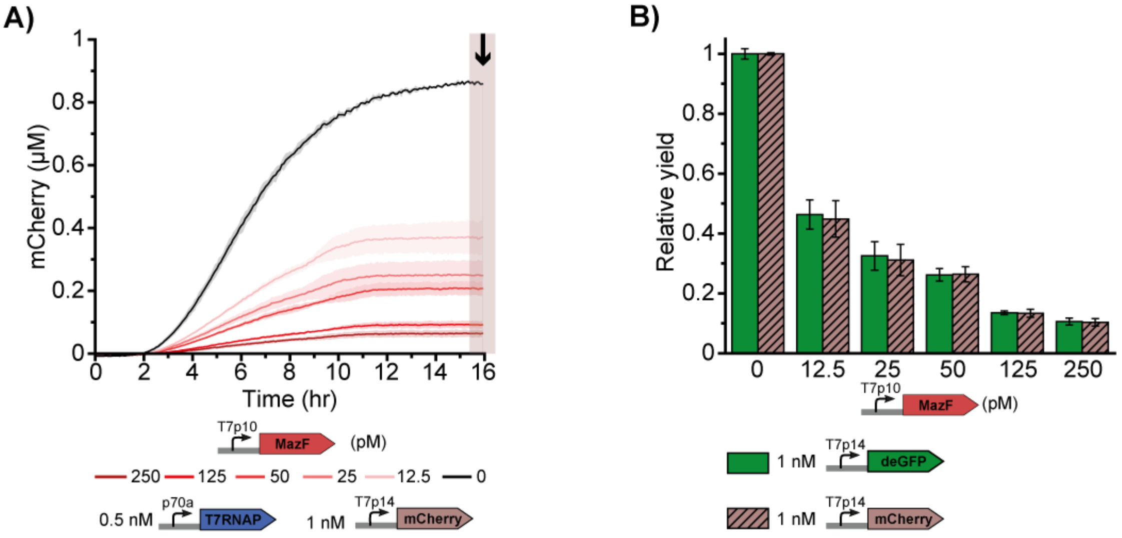


**Figure S6. Batch IVTT data showing the effect of MazF on mCherry expression. (A)** Expression of 1 nM T7p14-mCherry template (14 ACA sites) in combination with a range (0-250 pM) of T7p10-MazF template concentrations. The purple bar with arrow highlights the 16 h. timepoint at which the yields were calculated. **(B)** Comparison of the effect of a range of T7p10-MazF template concentrations on the expression yields at 16 h. (see Figure S7B and panel A) of 1 nM T7p14-deGFP (green) and 1 nM T7p14-mCherry (purple shaded) normalized to the 0 pM) T7p14-MazF condition.

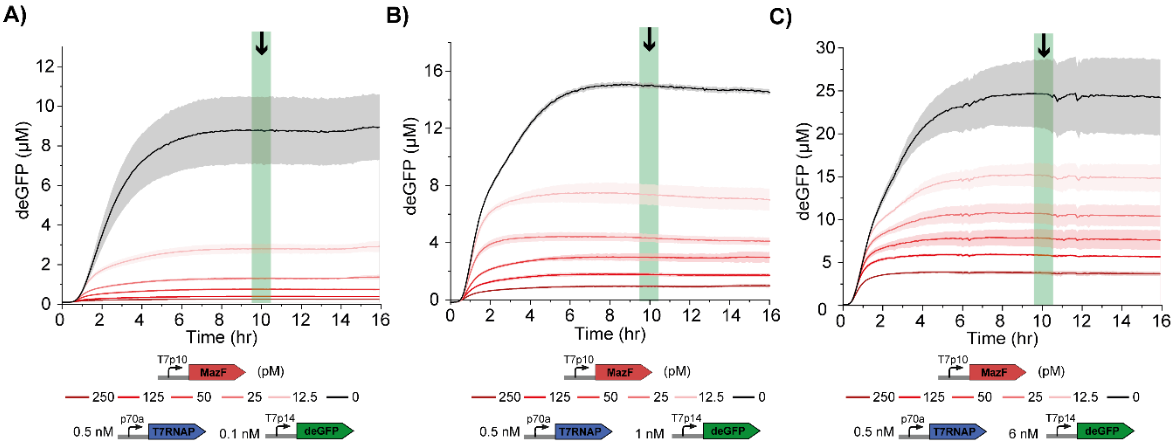


**Figure S7. Time courses of deGFP expression for conditions in Figure 3C.** Expression of 0.1 nM (**A**), 1 nM (**B**) or 6 nM (**C**) T7p14-deGFP template in combination with a range (0-250 pM) of T7p10-MazF template concentrations. The green bar represents the 10 h. timepoint at which the deGFP yields were analyzed for the heatmap in Figure 3C.

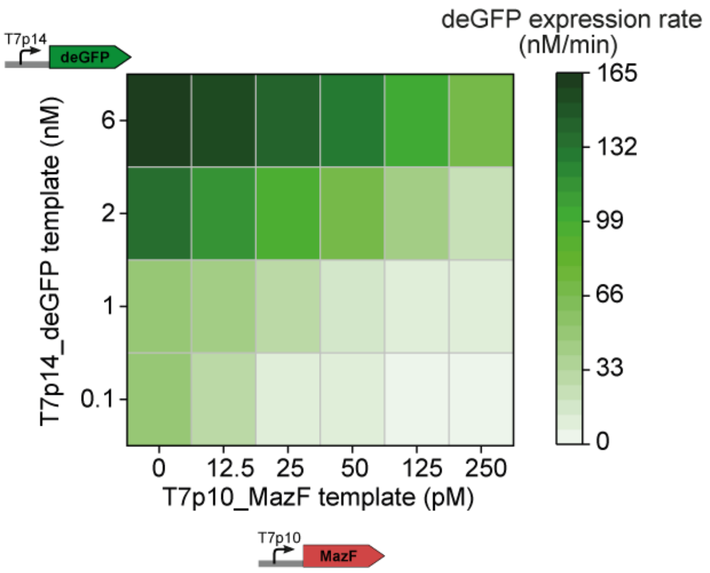


**Figure S8. Heatmap representing the maximum expression rates for the conditions in Figure 3C.** The maximum expression rates for a range of T7p10-MazF and T7p14-deGFP template concentrations. The maximum expression rates were calculated over a 30-minute time frame, as shown in Figure S2B.

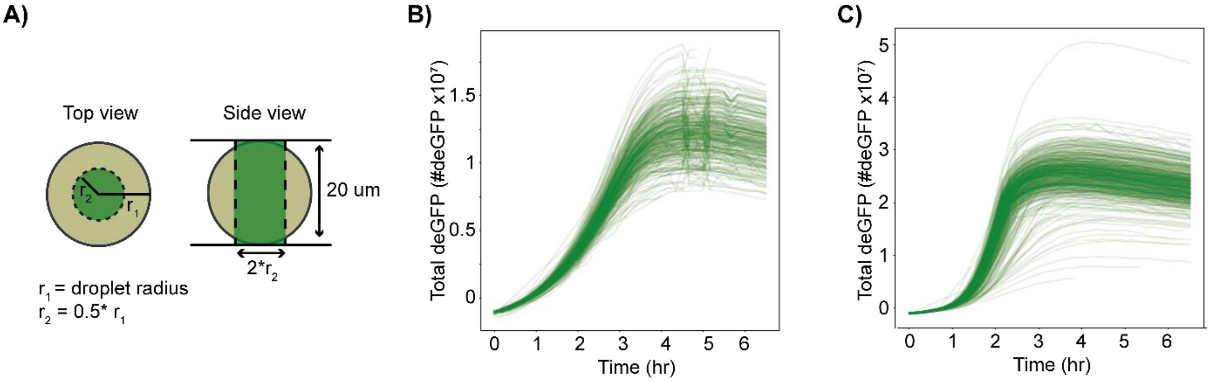


**Figure S9. Single droplet deGFP trajectories for *Equal template* and *Equal yield* experiments. (A)** Cartoon showing the reduced droplet area used to calculate the deGFP production. Only the pixels falling within the green circle (with a radius of half the total radius) were considered to calculate the droplet intensity. To calculate the volume of the droplet the green area was estimated to be a cylinder with an area given by the reduced radius and a height equaling the height of the collection chamber (20 µm). (**B-C**) Single droplet trajectories for the total deGFP production of the *Equal template (*1 nM T7p14-deGFP + 12.5 pM T7p10-MazF (B) and *Equal yield* (6 nM T7p14-deGFP + 12.5 pM T7p10-MazF) experiments (C). Only droplets that could be tracked for at least 10 timepoints are shown.

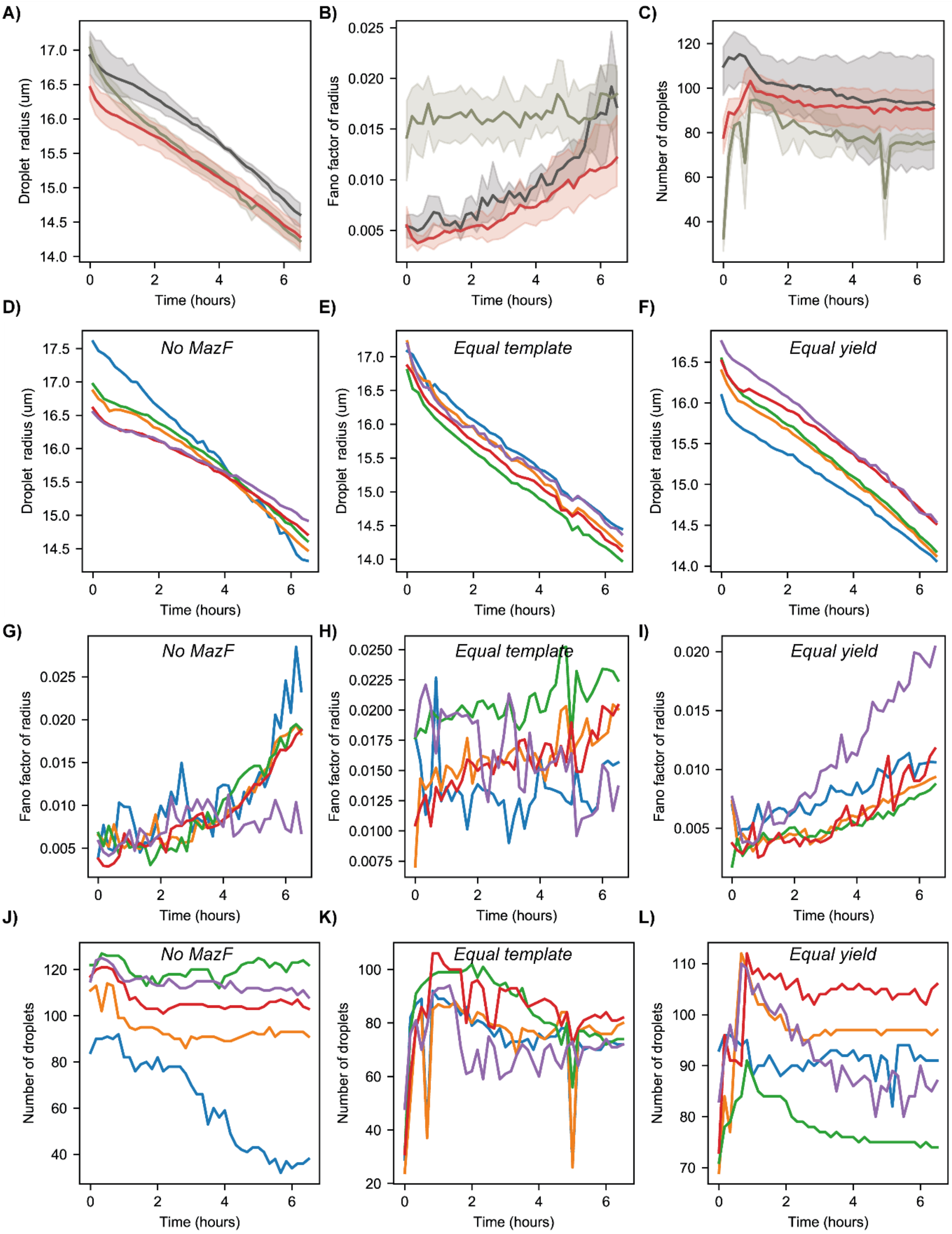


**Figure S10. Analysis of droplet characteristics per position.** Average droplet radius **(A)**, average Fano factor of the droplet radius **(B)** and average number of droplets **(C)** of the 5 positions for the 3 experiments in figure 4: *No MazF* (1 nM T7p14-deGFP template (black)), *Equal template* (1 nM T7p14-deGFP template + 12.5 pM T7p10-MazF template (yellow)), *Equal yield* (6 nM T7p14-deGFP template + 12.5 pM T7p10-MazF template (red)). The error envelope represents 1 standard deviation. Mean droplet radius **(D-F)**, Fano factor of droplet radius **(G-I)** and the number of analyzed droplets **(J-L)** per experiment over time. The left graphs (D,G,J) correspond to *No MazF*, the central graphs (E,H,K) corresponds to *Equal template* and the right graphs (F,I,L) correspond to the *Equal yield* experiment.

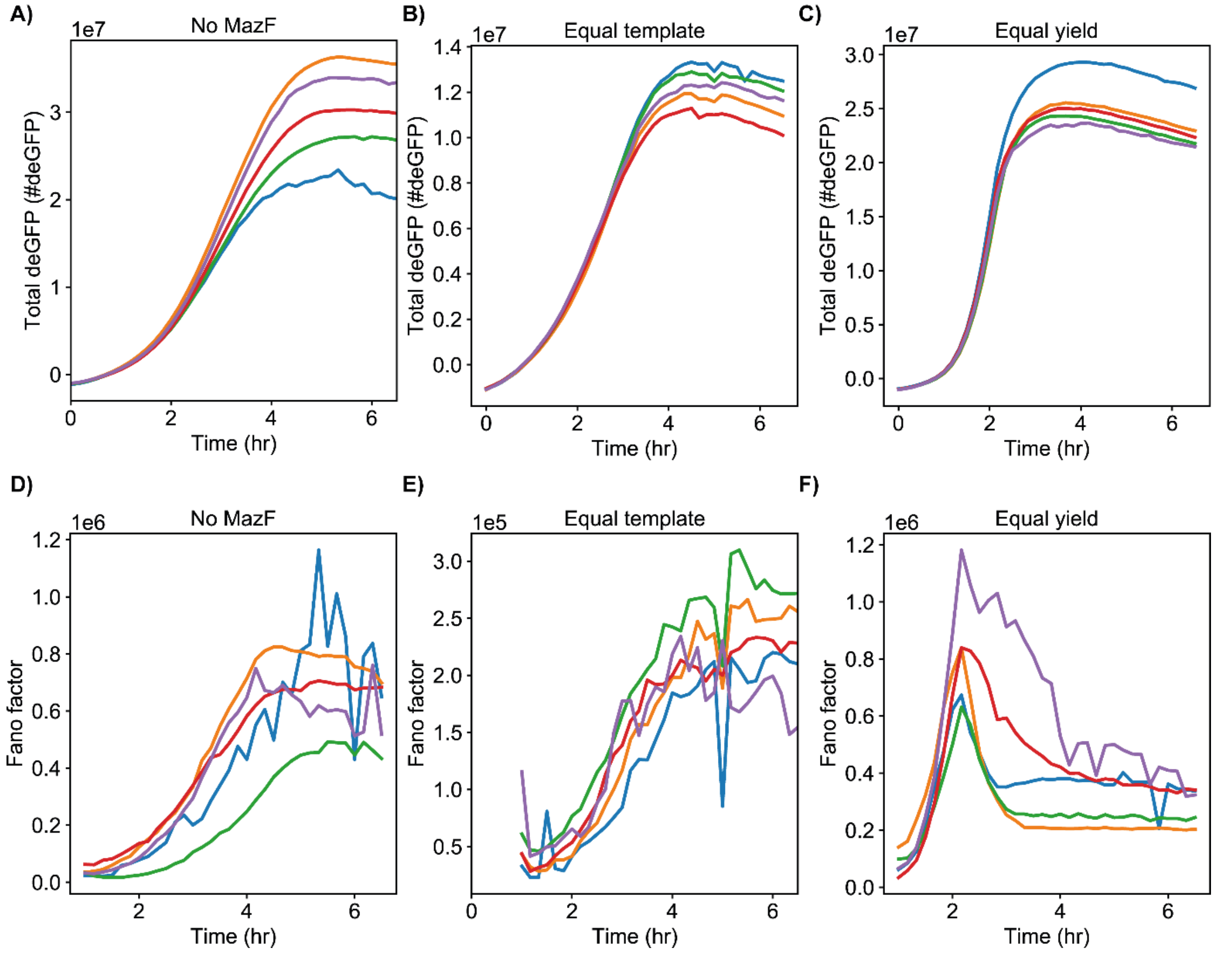


**Figure S11. Analysis of droplet intensities per position. (A-C)** Plots showing the total deGFP production per droplet (# deGFP molecules) over time per position from the experiments in Figure 4: *No MazF* (1 nM T7p14-deGFP template (black)), *Equal template* (1 nM T7p14-deGFP template + 12.5 pM T7p10-MazF template (yellow)), *Equal yield* (6 nM T7p14-deGFP template + 12.5 pM T7p10-MazF template (red)). **(D-F)** Fano factor of deGFP production over time for the same positions as in (**A-C**).

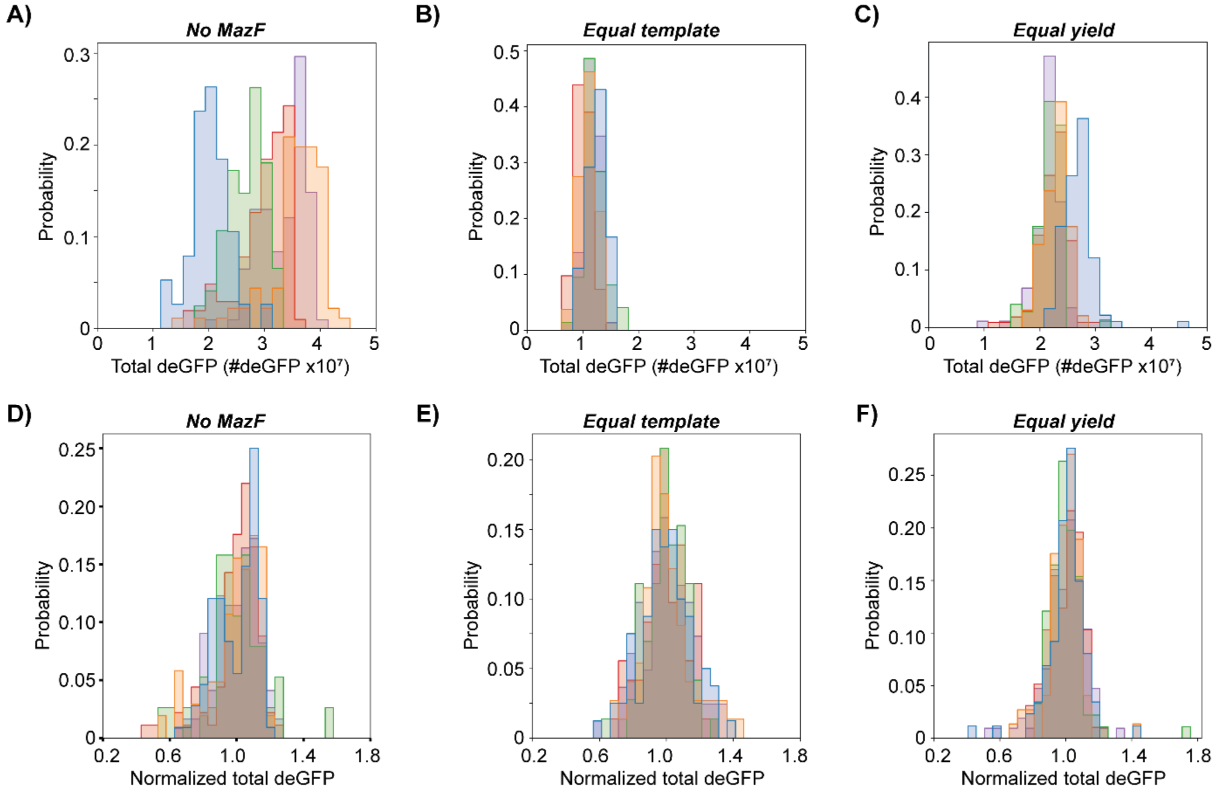


**Figure S12. Histograms showing the distribution of deGFP production yield per droplet after 6.5 hours. (A-C)** Probability distribution of the total deGFP production per droplet for the *No MazF* (A), *Equal template* (B), and *Equal yield* (C) conditions shown in Figure 4. Each of the 5 recorded positions are shown in a different color. (**D-F**) The same probability distributions as shown A-C, but normalized by position mean. The normalized total deGFP production distribution show overlap between the different positions indicating that the noise between the positions within the same experiment is comparable.

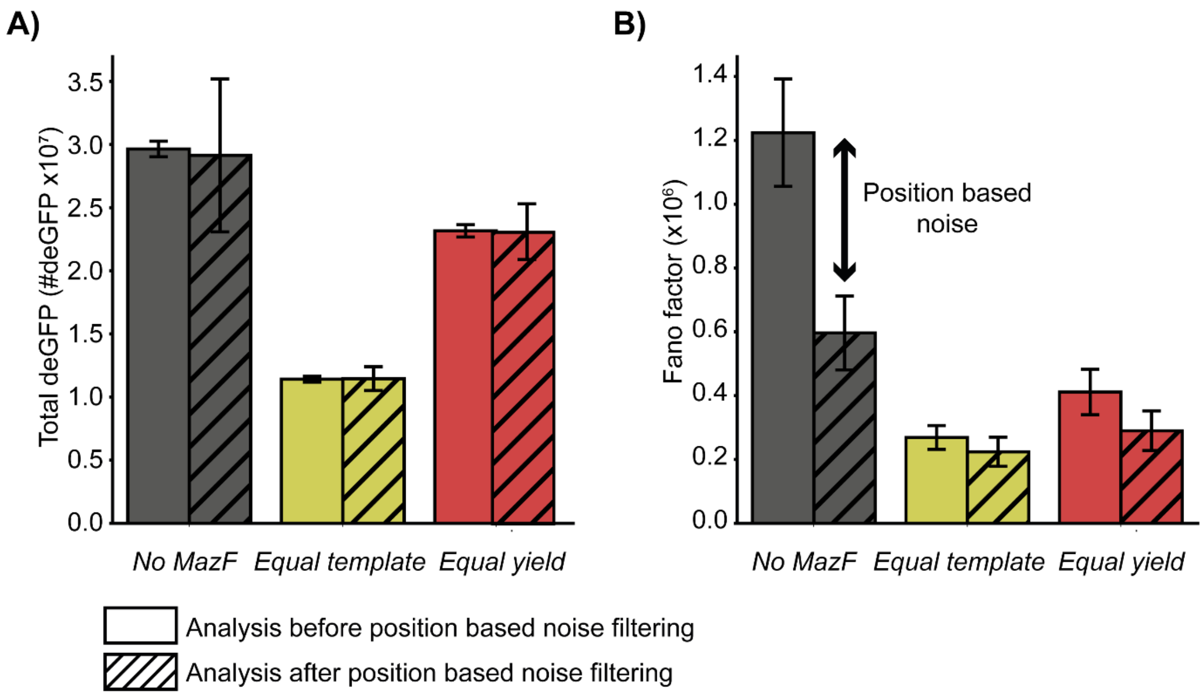


**Figure S13. Analysis per position removes position-based noise**. To account for the differences in deGFP production between different recorded positions (i.e., fields of view) within the same experiment, a position-based noise filter was applied (Supplementary methods). The final deGFP production of the experiments shown in Figure 4 were analyzed without (unshaded) and with (shaded) filtering the data per position. For the analysis without position information 5 subsets of droplets were created by selecting 20% of all droplets in each experiment at random and for the position based filtered data each position was analyzed separately. **(A)** The mean total deGFP production per droplet is unchanged by the position-based filtering step. **(B)** The Fano factor of the total deGFP production is decreased by the noise filtering step. The error bars represent the standard deviation between the analyzed subsets.

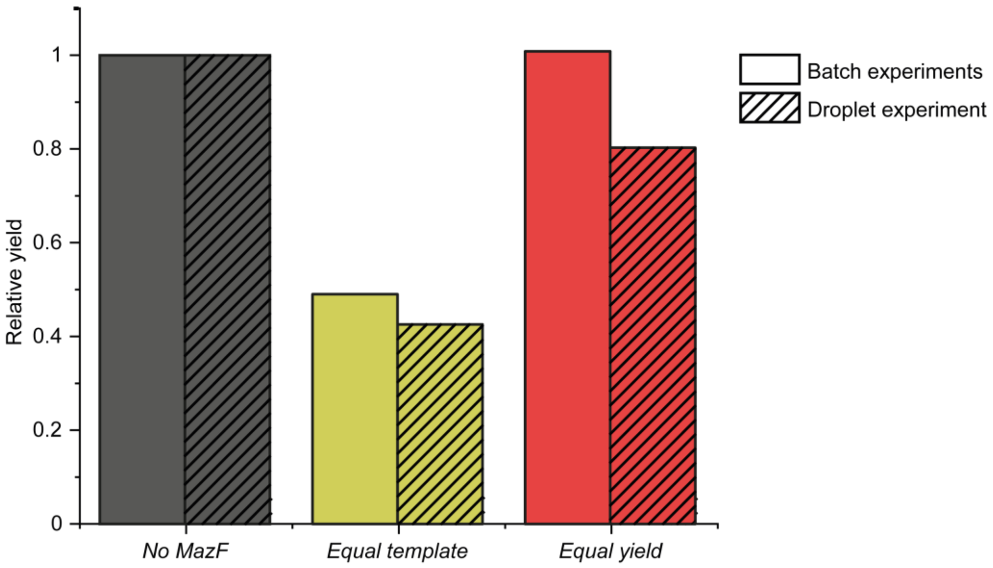


**Figure S14. Comparison of relative production yields between batch and droplet experiments.** Barplot showing the yields of the batch (open) and droplet (shaded) experiments: *No MazF* (1 nM T7p14-deGFP template (black)), *Equal template* (1 nM T7p14-deGFP template + 12.5 pM T7p10-MazF template (yellow)), *Equal yield* (6 nM T7p14-deGFP template + 12.5 pM T7p10-MazF template (red)). The yields were taken after 10 hours for batch reactions and after 6.5 hours for the droplet experiments and subsequently normalized to the yield of the *No MazF* experiment.

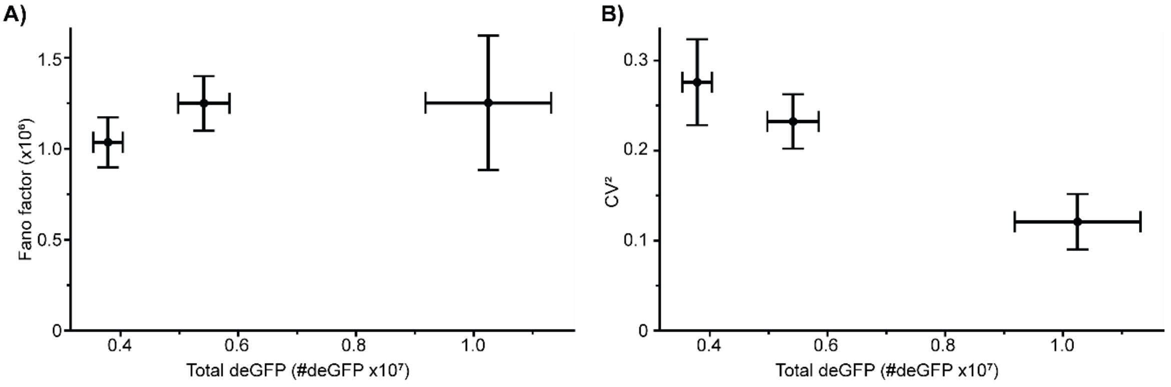


**Figure S15. Effect of total deGFP production on the Fano factor and CV$^2$.** To determine the effect of the total deGFP production on the noise measurement a range of T7p14-deGFP templates (0.6-1 nM) were expressed in droplets. **(A)** Scatterplot describing the relationship between the Fano factor ($\sigma^2/\mu$) and total deGFP production. (**B**) Scatterplot describing the relationship between the CV$^2$ ($\sigma^2/\mu^2$) and total deGFP production. The Fano factor shows no significant change with respect to deGFP yield, while the CV$^2$ increases for lower deGFP production yields. The error bars represent the standard deviation of 5 analyzed positions.

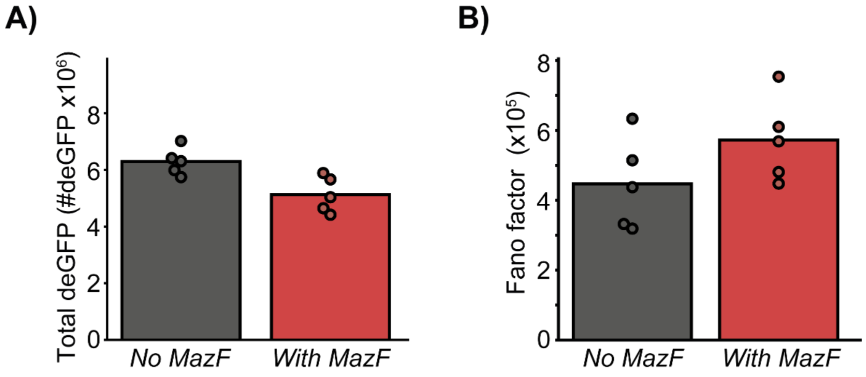


**Figure S16. The effect of MazF on the expression noise of the recoded T7p14-0ACAdeGFP template.** 0.5 nM p70a-T7RNAP and 1 nM T7p14-0ACAdeGFP were expressed in droplets without (*No MazF*) or with 125 pM T7p10-MazF template (*With MazF*). **(A)** The total deGFP production (after 6.5 hours) per droplet was slightly, but significantly, lower in the presence of the MazF template. **(B)** The Fano factor of the deGFP yield did not significantly differ between the two experiments.

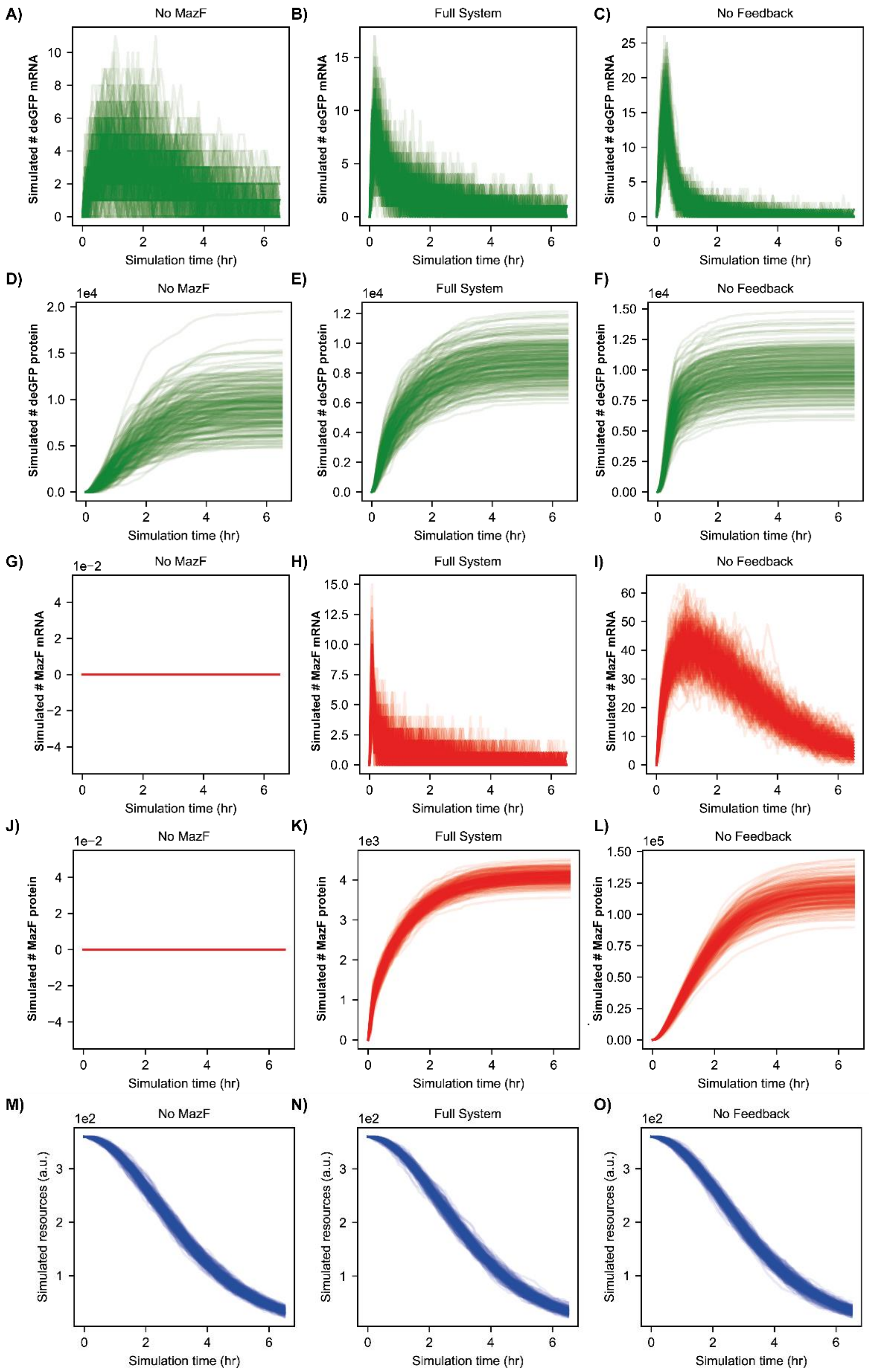


**Figure S17. Overview of individual trajectories from stochastic simulations.** Individual trajectories of the deGFP mRNA **(A-C)**, deGFP protein **(D-F)**, MazF mRNA **(G-I)**, MazF protein **(J-L)** and resources **(M-O).** The left column (A,D,G,J,M) shows the results for the *No MazF* model, the middle column (B,E,H,K,N) those of the *Full system* model and in the right column (C,F,I,L,O) the results of the *No Feedback* model are shown. 250 randomly selected traces are shown out of the full set of simulations.

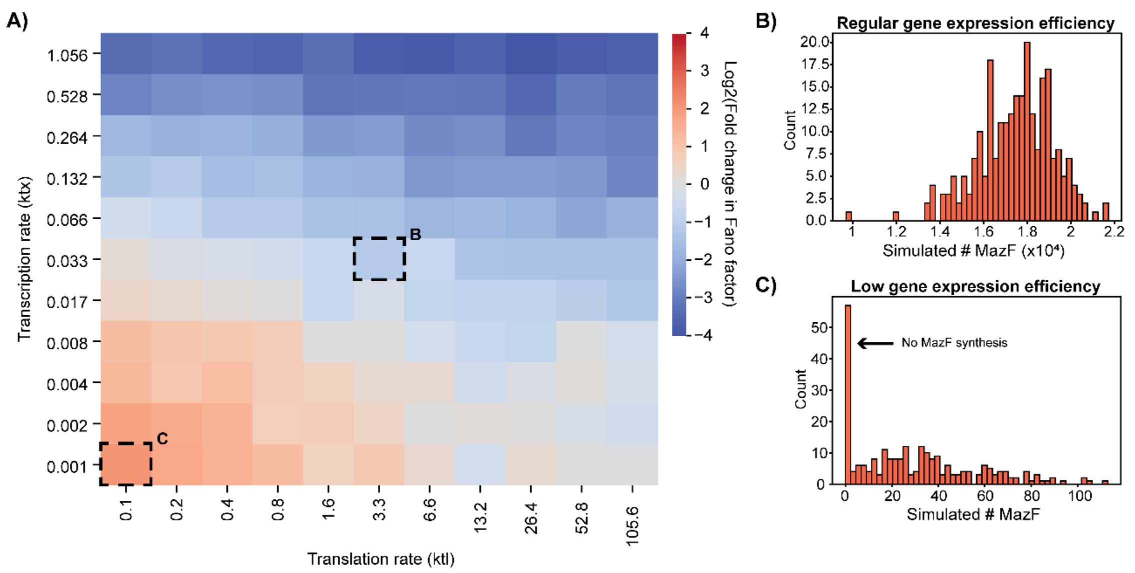


**Figure S18. Stochastic simulations of deGFP synthesis in presence or absence of MazF synthesis module for a range of transcription and translation rates.** **(A)** Heatmap of $Log_2$(Fold change) in Fano factor for a simulated population of droplets expressing deGFP in the presence of the MazF module versus the absence of the MazF module, after 2.5 hours of simulated expression. **(B-C)** Histogram of single-droplet values of expression for MazF protein after 1 hour of simulated expression of the systems with MazF synthesis module present, for parameter combinations highlighted in (A). The noise increase in the left-bottom corner of the heatmap can be explained by the large population of droplets expressing no MazF after 1hour. The parameter combination highlighted in box B is used for the simulations in Figure 5.

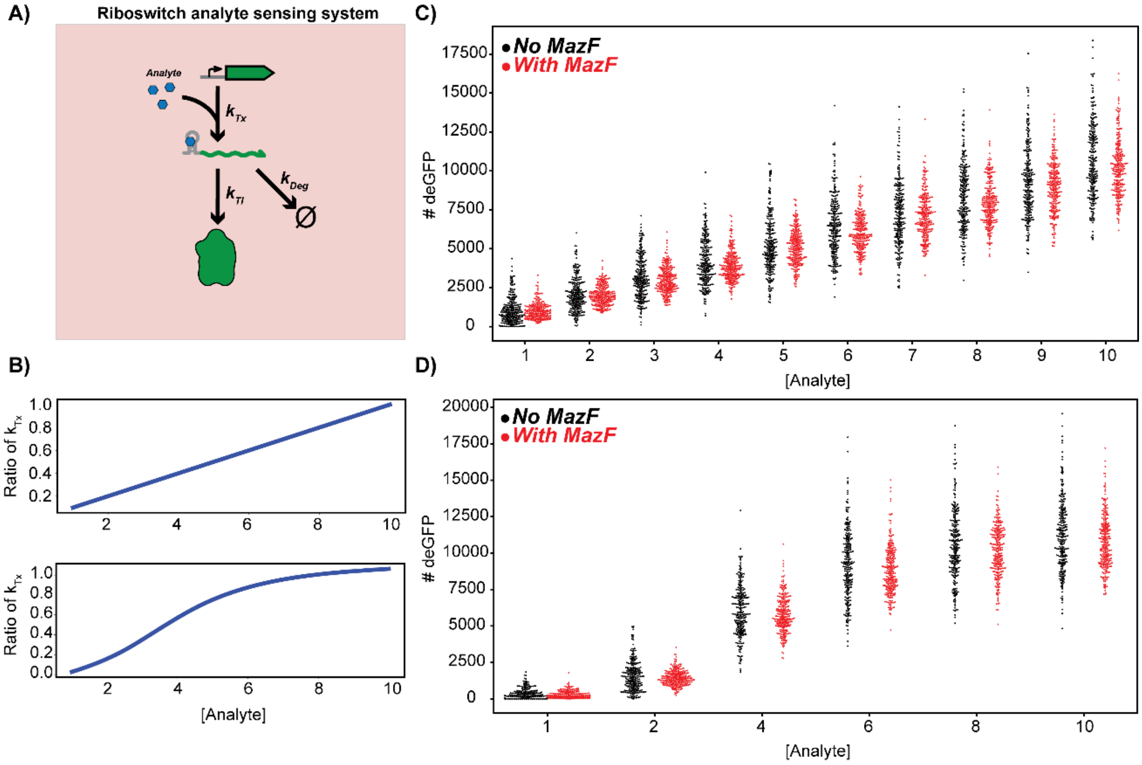


**Figure S19. Stochastic simulations of deGFP synthesis with a transcriptional riboswitch module. (A)** Schematic of an analyte-sensing deGFP synthesis system, based on a transcriptional riboswitch. An arbitrary analyte is required for transcription to proceed, thus the maximal $k_{Tx}$ is affected by the concentration of analyte. The module for MazF synthesis (not represented in this schematic), corresponds to the same structure depicted in Figure 5A. **(B)** Response curves between the ratio of maximal $k_{Tx}$ and the concentration of required analyte. Top; linear relationship established by: *Ratio of* $k_{Tx}$ *=([analyte]/[sat_analyte])* where *sat_analyte = 10* represents the concentration of analyte where the maximum $k_{Tx}$ is achieved. Bottom; sigmoidal relationship established by: *Ratio of* $k_{Tx}$ *= 1.13 + ((0.01+1.13) / (1 + (([analyte] / 3.96)*$^{2.98}$*)))*. **(C)** Single-droplet responses to a range of [Analyte] following a linear relationship depicted in B(top) in presence (red) and absence (black) of the MazF module. **(D)** Single-droplet response to range of [Analyte] following a sigmoidal relationship depicted in B (bottom) in presence (red) and absence (black) of the MazF module. In both models the droplet distribution shrinks in the presence of the MazF module showing the module's potential to improve biosensor performance in droplets.

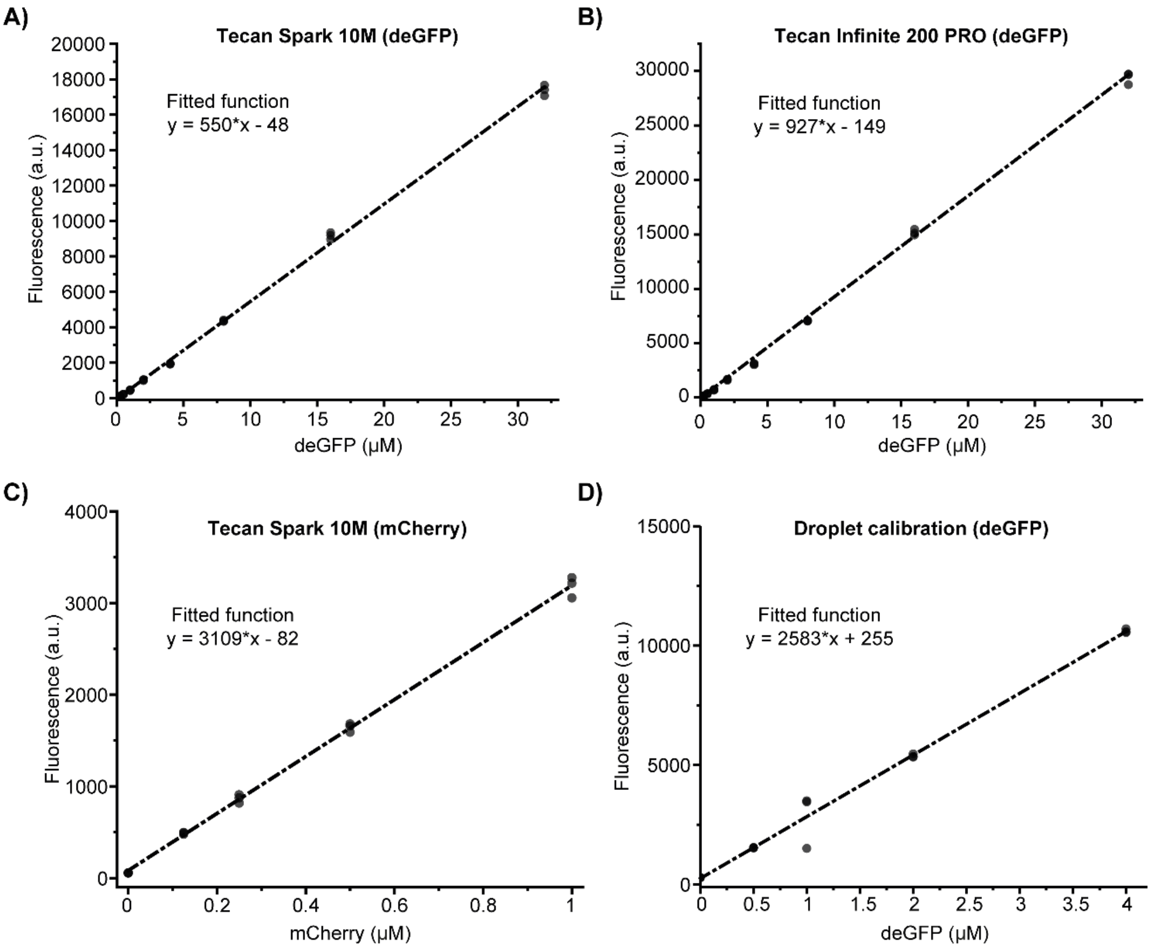


**Figure S20. Calibration curves for fluorescent proteins. (A)** deGFP calibration curve for the Spark 10M plate reader. The fluorescence was recorded with a gain of 40, an excitation wavelength of 485 nm and an emission wavelength of 535 nm. **(B)** deGFP calibration curve for the Infinite 200 PRO plate reader. The fluorescence was recorded with a gain of 100, an excitation wavelength of 488 nm and an emission wavelength of 520 nm. **(C)** mCherry calibration curve for the Spark 10M plate reader. The fluorescence was recorded with a gain of 80, an excitation wavelength of 560 nm and an emission wavelength of 620 nm. **(D)** Calibration curve for the microscope used in droplet experiments. Purified eGFP solutions were injected into the collection chambers and the fluorescence was recorded as described in the methods. The dots represent different z-positions. For the plate reader the fluorescence of known eGFP or mCherry concentrations were recorded in triplicates (dots) and the dashed lines show the linear fit functions (fitted using linear fit in Excel). $R^2$-values for all fits were larger than 0.998. A solution of purified eGFP was used for the calibration curves, but since eGFP has the same spectroscopic properties as deGFP in the rest of the manuscript deGFP is used for all axis[6].

| **Name** | Description | Source |
|---|---|---|
| p70a-T7RNAP | pTXTL plasmid used for the expression of the T7-RNA polymerase enzyme in IVTT. | ArborBiosciences |
| T7p10-MazF | pET23b-plasmid used for the expression of the MazF enzyme in IVTT. | This work |
| T7p14-deGFP | pTXTL-plasmid used for the expression of deGFP in IVTT . | ArborBiosciences |
| T7p10-17ACAdeGFP | pTXTL-plasmid used for the production of recoded deGFP mRNA (seventeen ACA sites) | This work |
| T7p10-0ACAdeGFP | pTXTL-plasmid used for the production of recoded deGFP mRNA (zero ACA sites) | This work |
| T7p10-1ACAdeGFP | pTXTL-plasmid used for the production of recoded deGFP mRNA (one ACA sites) | This work |
| T7p10-2ACAdeGFP | pTXTL-plasmid used for the production of recoded deGFP mRNA (two ACA sites) | This work |
| T7p14-0ACAdeGFP | pTXTL-plasmid used for the expression of recoded deGFP (zero ACA sites) in IVTT. | This work |
| T7p14-mCherry-Spinach2 | pRset5d-pasmid used for the expression of mCherry reporter protein in IVTT. | This work |

**Table S1. Table of templates used in this work.** Table summarizing the templates used in this work. The plasmids from ArborBiosciences were previously described in [7]. For the plasmids constructed in this work the gene seqeunces are sumarized in table S2 and the sources of the fragments in Table S4.

## T7p10-MazF (11 ACA sites)

taatacgactcactatagggAGACCACAACGGTTTCCCTCTAGAAATAATTTTGTTTAACTTTAAGAAGGAGATATACA
TATGGTAAGCCGATACGTACCCGATATGGGCGATCTGATTTGGGTTGATTTTGACCCGACAAAAGGTAGCG
AGCAAGCTGGACATCGTCCAGCTGTTGTCCTGAGTCCTTTCATGTACAACAACAAAACAGGTATGTGTCTGT
GTGTTCCTTGTACAACGCAATCAAAAGGATATCCGTTCGAAGTTGTTTTATCCGGTCAGGAACGTGATGGCG
TAGCGTTAGCTGATCAGGTAAAAAGTATCGCCTGGCGGGCAAGAGGAGCAACGAAGAAAGGAACAGTTGC
CCCAGAGGAATTACAACTCATTAAAGCCAAAATTAACGTACTGATTGGGTAG

## T7p10-17ACAdeGFP

taatacgactcactatagggGGCAGAATAATTTTGTTTAACTTTAAGAAGGAGATATACAATGGAGCTTTTCACTGGCG
TTGTTCCCATCCTGGTCGAGCTGGACGGCGACGTAAACGGCC**ACA**AGTTCAGCGTGTCCGGCGAGGGCGAG
GGCGATGCCACCTACGGCAAGCTGACCCTGAAGTTCATCTGCACCACCGGCAAGCTGCCCGTGCCCTGGCC
CACCCTCGTGACCACCCTGACCTACGGCGTGCAGTGCTTCAGCCGCTACCCCGACC**ACA**TGAAGCAGCACG
ACTTCTTCAAGTCCGCCATGCCCGAAGGCTACGTCCAGGAGCGCACCATCTTCTTCAAGGACGACGGCAAC
T**ACA**AGACCCGCGCCGAGGTGAAGTTCGAGGGCG**ACA**CCCTGGTGAACCGCATCGAGCTGAAGGGCATCG
ACTTCAAGGAGGACGGCA**ACA**TCCTGGGGC**ACA**AGCTGGAGT**ACA**ACT**ACAACA**GCC**ACA**ACGTCTATAT
CATGGCCG**ACA**AGCAGAAGAACGGCATCAAGGTGAACTTCAAGATCCGCC**ACAACA**TCGAGGACGGCAGC
GTGCAGCTCGCCGACCACTACCAGCAGA**ACA**CCCCCATCGGCGACGGCCCCGTGCTGCTGCCCG**ACA**ACCA
CTACCTGAGCACCCAGTCCGCCCTGAGCAAAGACCCCAACGAGAAGCGCGATC**ACA**TGGTCCTGCTGGAGT
TCGTGACCGCCGCCGGGATCTAA

## T7p10-0ACAdeGFP

taatacgactcactatagggCAGAATAATTTTGTTTAACTTTAAGAAGGAGATATAGAATGGAGCTTTTCACTGGCGTT
GTTCCCATCCTGGTCGAGCTGGACGGCGACGTAAACGGCC**ATA**AGTTCAGCGTGTCCGGCGAGGGCGAGG
GCGATGCCACCTACGGCAAGCTGACCCTGAAGTTCATCTGCACCACCGGCAAGCTGCCCGTGCCCTGGCCC
ACCCTCGTGACCACCCTGACCTACGGCGTGCAGTGCTTCAGCCGCTACCCCGACC**ATA**TGAAGCAGCACGA
CTTCTTCAAGTCCGCCATGCCCGAAGGCTACGTCCAGGAGCGCACCATCTTCTTCAAGGACGACGGCAACT
**ATA**AGACCCGCGCCGAGGTGAAGTTCGAGGGCG**ATA**CCCTGGTGAACCGCATCGAGCTGAAGGGCATCGA
CTTCAAGGAGGACGGCAATATCCTGGGGC**ATA**AGCTGGAGT**ATA**ACT**ATAATA**GCC**ATA**ACGTCTATATC
ATGGCCG**ATA**AGCAGAAGAACGGCATCAAGGTGAACTTCAAGATCCGCC**ATAATA**TCGAGGACGGCAGCG
TGCAGCTCGCCGACCACTACCAGCAGA**ATA**CCCCCATCGGCGACGGCCCCGTGCTGCTGCCCG**ATA**ACCAC
TACCTGAGCACCCAGTCCGCCCTGAGCAAAGACCCCAACGAGAAGCGCGATC**ATA**TGGTCCTGCTGGAGTT
CGTGACCGCCGCCGGGATCTAA

## T7p10-1ACAdeGFP

taatacgactcactatagggCAGAATAATTTTGTTTAACTTTAAGAAGGAGATATAGAATGGAGCTTTTCACTGGCGTT
GTTCCCATCCTGGTCGAGCTGGACGGCGACGTAAACGGCC**ATA**AGTTCAGCGTGTCCGGCGAGGGCGAGG
GCGATGCCACCTACGGCAAGCTGACCCTGAAGTTCATCTGCACCACCGGCAAGCTGCCCGTGCCCTGGCCC
ACCCTCGTGACCACCCTGACCTACGGCGTGCAGTGCTTCAGCCGCTACCCCGACC**ATA**TGAAGCAGCACGA
CTTCTTCAAGTCCGCCATGCCCGAAGGCTACGTCCAGGAGCGCACCATCTTCTTCAAGGACGACGGCAACT
**ATA**AGACCCGCGCCGAGGTGAAGTTCGAGGGCG**ATA**CCCTGGTGAACCGCATCGAGCTGAAGGGCATCGA
CTTCAAGGAGGACGGCAATATCCTGGGGC**ATA**AGCTGGAGT**ATA**ACT**ATAATA**GCC**ATA**ACGTCTATATC
ATGGCCG**ATA**AGCAGAAGAACGGCATCAAGGTGAACTTCAAGATCCGCC**ATAATA**TCGAGGACGGCAGCG
TGCAGCTCGCCGACCACTACCAGCAGA**ACA**CCCCCATCGGCGACGGCCCCGTGCTGCTGCCCG**ATA**ACCAC
TACCTGAGCACCCAGTCCGCCCTGAGCAAAGACCCCAACGAGAAGCGCGATC**ATA**TGGTCCTGCTGGAGTT
CGTGACCGCCGCCGGGATCTAA

| **T7p10-2ACAdeGFP** |
|---|
| taatacgactcactatagggCAGAATAATTTTGTTTAACTTTAAGAAGGAGATATAGAATGGAGCTTTTCACTGGCGTT<br>GTTCCCATCCTGGTCGAGCTGGACGGCGACGTAAACGGCCATAAGTTCAGCGTGTCCGGCGAGGGCGAGG<br>GCGATGCCACCTACGGCAAGCTGACCCTGAAGTTCATCTGCACCACCGGCAAGCTGCCCGTGCCCTGGCCC<br>ACCCTCGTGACCACCCTGACCTACGGCGTGCAGTGCTTCAGCCGCTACCCCGACCATATGAAGCAGCACGA<br>CTTCTTCAAGTCCGCCATGCCCGAAGGCTACGTCCAGGAGCGCACCATCTTCTTCAAGGACGACGGCAACT<br>ATAAGACCCGCGCCGAGGTGAAGTTCGAGGGCGATACCCTGGTGAACCGCATCGAGCTGAAGGGCATCGA<br>CTTCAAGGAGGACGGCAATATCCTGGGGCATAAGCTGGAGTATAACTATAATAGCCATAACGTCTATATC<br>ATGGCCGATAAGCAGAAGAACGGCATCAAGGTGAACTTCAAGATCCGCCACAACATCGAGGACGGCAGCG<br>TGCAGCTCGCCGACCACTACCAGCAGAATACCCCCATCGGCGACGGCCCCGTGCTGCTGCCCGATAACCAC<br>TACCTGAGCACCCAGTCCGCCCTGAGCAAAGACCCCAACGAGAAGCGCGATCATATGGTCCTGCTGGAGTT<br>CGTGACCGCCGCCGGGATCTAA |
| **T7p14-0ACAdeGFP** |
| taatacgactcactatagggAGACCAGAACGGTTTCCCTCTAGAAATAATTTTGTTTAACTTTAAGAAGGAGATATAGA<br>ATGGAGCTTTTCACTGGCGTTGTTCCCATCCTGGTCGAGCTGGACGGCGACGTAAACGGCCATAAGTTCAG<br>CGTGTCCGGCGAGGGCGAGGGCGATGCCACCTACGGCAAGCTGACCCTGAAGTTCATCTGCACCACCGGCA<br>AGCTGCCCGTGCCCTGGCCCACCCTCGTGACCACCCTGACCTACGGCGTGCAGTGCTTCAGCCGCTACCCCG<br>ACCATATGAAGCAGCACGACTTCTTCAAGTCCGCCATGCCCGAAGGCTACGTCCAGGAGCGCACCATCTTC<br>TTCAAGGACGACGGCAACTATAAGACCCGCGCCGAGGTGAAGTTCGAGGGCGATACCCTGGTGAACCGCA<br>TCGAGCTGAAGGGCATCGACTTCAAGGAGGACGGCAATATCCTGGGGCATAAGCTGGAGTATAACTATAA<br>TAGCCATAACGTCTATATCATGGCCGATAAGCAGAAGAACGGCATCAAGGTGAACTTCAAGATCCGCCAT<br>AATATCGAGGACGGCAGCGTGCAGCTCGCCGACCACTACCAGCAGAATACCCCCATCGGCGACGGCCCCG<br>TGCTGCTGCCCGATAACCACTACCTGAGCACCCAGTCCGCCCTGAGCAAAGACCCCAACGAGAAGCGCGAT<br>CATATGGTCCTGCTGGAGTTCGTGACCGCCGCCGGGATCTAA |
| **T7p14-mCherry (14 ACA sites)** |
| taatacgactcactatagggAGACCACAACGGTTTCCCTCTAGAAATAATTTTGTTTAACTTTAAGAAGGAGATATACC<br>ATGGTGAGCAAGGGCGAAGAAGATAACATGGCCATCATCAAGGAGTTCATGCGCTTCAAGGTGCACATGG<br>AGGGCTCCGTGAACGGCCACGAGTTCGAGATCGAGGGCGAGGGCGAGGGCCGCCCCTACGAGGGCACCCA<br>GACCGCCAAGCTGAAGGTGACCAAGGGTGGCCCCCTGCCCTTCGCCTGGGACATCCTGTCCCCTCAGTTCA<br>TGTACGGCTCCAAGGCCTACGTGAAGCACCCCGCCGACATCCCCGACTACTTGAAGCTGTCCTTCCCCGAG<br>GGCTTCAAGTGGGAGCGCGTGATGAACTTCGAGGACGGCGGCGTGGTGACCGTGACCCAGGACTCCTCCCT<br>GCAGGACGGCGAGTTCATCTACAAGGTGAAGCTGCGCGGCACCAACTTCCCCTCCGACGGCCCCGTAATGC<br>AGAAGAAGACCATGGGCTGGGAGGCCTCCTCCGAGCGGATGTACCCCGAGGACGGCGCCCTGAAGGGCGA<br>GATCAAGCAGAGGCTGAAGCTGAAGGACGGCGGCCACTACGACGCTGAGGTCAAGACCACCTACAAGGCC<br>AAGAAGCCCGTGCAGCTGCCCGGCGCCTACAACGTCAACATCAAGTTGGACATCACCTCCCACAACGAGG<br>ACTACACCATCGTGGAACAGTACGAACGCGCCGAGGGCCGCCACTCCACCGGCGGCATGGACGAGCTGTA<br>CAAGTAA |

**Table S2. Table of gene sequences for the plasmids constructed in this work.** DNA sequences are shown from the promotor (small case) to the stop codon of the gene coding sequence. The complete coding sequences is underlined. The (recoded) ACA sites are highlighted in red.

Fitted exponential decay function: $\mathbf{y = a*e^{b*t}}$

| Experiment | Half-life (min) | Adjusted $R^2$ | a | b ($min^{-1}$) |
|---|---|---|---|---|
| **17ACA_(-)MazF** | 44 | 0.852 | 0.912 ± 0.042 | -0.016 ± 0.003 |
| **17ACA_(+)MazF** | 2.4 | 0.999 | 1.000 ± 0.011 | -0.284 ± 0.006 |
| **0ACA_(-)MazF** | 63 | 0.820 | 1.018 ± 0.038 | -0.011 ± 0.002 |
| **0ACA_(+)MazF** | 20 | 0.915 | 0.911 ± 0.048 | -0.034 ± 0.006 |
| **1ACA_(-)MazF** | 87 | 0.650 | 0.910 ± 0.039 | -0.008 ± 0.002 |
| **1ACA_(+)MazF** | 9.9 | 0.971 | 0.943 ± 0.040 | -0.070 ± 0.007 |
| **2ACA_(-)MazF** | 173 | 0.541 | 1.060 ± 0.035 | -0.004 ± 0.002 |
| **2ACA_(+)MazF** | 7.5 | 0.931 | 0.908 ± 0.063 | -0.093 ± 0.014 |
| **MazF_(-)MazF** | 30 | 0.915 | 1.075 ± 0.050 | -0.023 ± 0.004 |
| **MazF_(+)MazF** | 5.8 | 0.887 | 0.921 ± 0.090 | -0.120 ± 0.026 |

**Table S3. Exponential decay parameters of mRNA degradation experiments.** An exponential decay function ($y= a*e^{bt}$) was fitted to data represented in figure 2C. The values for parameters a and b are shown with the standard error and the adjusted $R^2$. From the value of parameter b, the half-life is calculated.

| Fragment | Source |
|---|---|
| pET-23b | Novagen |
| MazF | IDT |
| pTXTL-Backbone | p70a-deGFP |
| 5’-T7p10 | Unpublished plasmid, a combination of the 5’protective region from pTXTL-p70a-deGFP and T7p10 from pET23b-MazF |
| T7p14 | IDT, recoded version of T7p14 |
| UTR1 | p70a-deGFP |
| UTR1-0ACA | IDT, recoded version of UTR1 |
| deGFP | pTXTL-p70a-deGFP |
| 0ACAdeGFP | IDT, recoded version of deGFP |
| tT7-3’ | Unpublished plasmid, a combination of the 3’protective region from p70a-deGFP and tT7 terminator from pET23b-MazF |
| pRSET5d | pRSET5d-Spi2 |
| mCherry | IDT |
| Spinach2 | pRSET5d-Spi2 |

**Table S4. Origins of DNA fragments used for cloning**. The pTXTL-p70aGFP plasmid was ordered from ArborBiosciences and previously reported[7]. The fragments ordered from IDT were ordered as a double stranded gblock (MazF, 0ACAdeGFP, and mCherry) or single stranded ultramer (UTR1-0ACA, and T7p14). The 5’-T7p10 and tT7-3’ were amplified from a plasmid constructed for a different work (unpublished), but their sequence is a combination of the sequences described in the sources. The pRSET5d-Spi2 plasmid was constructed in our lab in earlier work.[8]

| Parameter | Value | Description | Equation |
|---|---|---|---|
| *DNA* | 6 or 1 | deGFP template concentration | 1 |
| $DNA_{mF}$ | 0.0125 | MazF template concentration | 4 |
| $k_{tx}$ | 0.033 | deGFP transcription | 1 |
| $k_{tx\_mF}$ | 16.5 | MazF  transcription | 4 |
| $k_{tl}$ | 3.3 | deGFP and MazF translation | 2 and 5 |
| $k_{deg}$ | 3 (if MazF = 0)<br>0.0017 (if MazF>0) | deGFP mRNA degradation | 1 |
| $k_{deg\_mF}$ | 3 (if MazF = 0)<br>0.00033 (if MazF>0) | MazF mRNA degradation | 4 |
| $k_{dep}$ | 0.00013 | Rate of resource depletion | 3 |
| *Res* | 360 | Initial resources | 3 |

**Table S5. Input parameters used for stochastic simulations.**

## References


1. Hu, X.;  van Sluijs, B.;  Garcia Blay, O.;  Stepanov, Y.;  Rietrae, K.;  Huck, W.; Hansen, M. M. K., ARTseq-FISH reveals position-dependent fate decisions driven by cell cycle changes. *bioRxiv* **2022**, 2022.09.14.507902.
2. Kaehler, B. G. R. a. A., *Learning Opencv : Computer Vision with the Opencv Library*. 1st ed. ed.; Sebastopol CA: O'Reilly: 2008.
3. Pedregosa, F. a. V., Ga, Scikit-learn: Machine learning in Python. *Journal of machine learning research* **2011,** *12* (Oct), 2825--2830.
4. McKinney, W. a. o., *Data structures for statistical computing in python*. 2010; Vol. 445.
5. Hunter, J. D., Matplotlib: A 2D Graphics Environment. *Computing in Science & Engineering* **2007,** *9* (3), 90-95.
6. Shin, J.; Noireaux, V., An E. coli Cell-Free Expression Toolbox: Application to Synthetic Gene Circuits and Artificial Cells. *ACS Synth. Biol.* **2012,** *1* (1), 29-41.
7. Garamella, J.;  Marshall, R.;  Rustad, M.; Noireaux, V., The All E. coli TX-TL Toolbox 2.0: A Platform for Cell-Free Synthetic Biology. *ACS Synth Biol* **2016,** *5* (4), 344-55.
8. Deng, N. N.;  Yelleswarapu, M.;  Zheng, L.; Huck, W. T., Microfluidic Assembly of Monodisperse Vesosomes as Artificial Cell Models. *J. Am. Chem. Soc.* **2017,** *139* (2), 587-590.
